# Complex effects of cooperative behavior on authorized remanufacturing supply chain decisions under subsidies

**DOI:** 10.1371/journal.pone.0291940

**Published:** 2023-09-21

**Authors:** Ling Zhang, Zheng Zhang

**Affiliations:** Business School, University of Shanghai for Science and Technology, Shanghai, China; Sichuan Agricultural University, CHINA

## Abstract

In this study, the dynamic effects of new product supply chain cooperation behavior on optimal government subsidies and supply chain decision-making are studied by establishing a nonlinear discrete inventory decision system; In this system, the government subsidizes authorized remanufacturers to promote remanufacturing, and cooperative behavior exists in the supply chain of new products. The research method is modeling and simulation of a supply chain system based on nonlinear system dynamics theory. The complexity analysis includes the stability analysis of the decision system, the path of the system into chaos, the change of entropy of the system and the performance in chaos system. Our findings indicate that the optimal government subsidy in the cooperative model is lower than that in the non-cooperative model. Consumer surplus is the main reason for the subsidy difference between the two models. In comparison with the cooperative supply chain, the stability of the non-cooperative supply chain is more easily affected by government subsidies. Further, the market is more likely to enter chaos due to improper adjustment of the new products’ inventory with cooperative behavior in the supply chain of new products. When the system enters chaos, the new product supply chain’s profit in the cooperative system is more likely to be far lower than the equilibrium profit. This study provides a theoretical reference for supply chain inventory management and government subsidy remanufacturer decision-making from the perspective of dynamic systems science.

## 1. Introduction

The circular economy contributes to sustainable social and economic development by reducing the consumption of resources and the generation of waste. The significance of the circular economy lies in the effective recovery of waste products and reuse of surplus value. Remanufacturing is an essential component of circular economy. Many companies worldwide have entered the field of remanufacturing because of its economical and environmentally friendly characteristics. Some examples are Hewlett Packard (HP), Apple, Kodak, Xerox, and Canon. Remanufacturing is the process of recycling used products, reprocessing them to obtain products with the same performance as the new products, and returning them again to the market [1].

At present, the theoretical research on remanufacturing closed-loop supply chain decision-making has two economic structures. First of all, the closed-loop supply chain under the remanufacturing of the original manufacturer does not carry out the differential pricing of new and remanufactured products, and the market stability is relatively high. Therefore, the selection of recycling channel, recovery rate, channel intrusion and other issues are mainly considered in the closed-loop supply chain under the remanufacturing of the original manufacturer [2, 3]. Secondly, research on decision-making in markets composed of original product supply chains and independent third-party remanufacturing supply chains has been extensive [4–6]. The current study includes waste product recycling channel management [7–9], choice of remanufacturing production method [10–12], impact of remanufactured products on the new product market, and influence of remanufacturing on new product decision [13–15]. Comprehensive analysis of the existing theoretical research, few studies have considered the impact of original product supply chain cooperative behavior on authorized remanufacturers. A market structure composed of a cooperative new product supply chain and third-party remanufacturing supply chain already exists in the economic system. For example, China’s JD.com retailer and South Korea’s Samsung Group signed a 2022 cooperative agreement to conduct in-depth precision marketing. Simultaneously, Samsung provided official remanufacturing authorization to third-party remanufacturing organizations. And as remanufacturing becomes more mature, independent remanufacturers will emerge, and the conflict between the original product and the third-party remanufactured product in the market is becoming increasingly apparent. The production mode, sales channel and consumer acceptance of third-party remanufacturing products all affect the development of remanufacturing [16]. However, the stability of market decision in remanufacturing closed-loop supply chain is rarely considered.

Government subsidies are also a driving force to support companies in recycling and remanufacturing. In order to promote the development of a circular economy, provincial governments have introduced a series of relevant policies to encourage the development of remanufacturing industry, including subsidies, old for new, industrial park construction. In February 2022, the U.S. Department of Energy announced it would provide approximately $60 million to fund research, development, and demonstration of battery recycling and reuse applications for electric vehicles. Countries such as the UK, India and China have implemented policies related to subsidies for remanufacturing [17]. Zhang et al. [18] studied the influence of government subsidies on the decision-making of a closed-loop supply chain with unit authorized remanufacturing under carbon tax policy. Research has found that when government subsidies are set in an appropriate range, subsidies are beneficial to the profits of the entire supply chain, social welfare, consumer surplus and the environment. The unit authorization fee was reduced after government subsidies. Government research on remanufacturing supply chain subsidies mainly focuses on determining optimal subsidies, the impact of subsidies on product price, environment, social welfare and consumer surplus, and the relationship between subsidies and authorized costs. However, few studies have considered the determination of government subsidies in remanufacturing supply chains with different economic structures and the dynamic effects of subsidies on supply chain decision-making.

The integration of nonlinear dynamics and management science makes us realize the nonlinear relationship that may exist in the market decision-making of remanufacturing closed-loop supply chain. And the influence of nonlinear relationships and related factors will lead to market imbalance into chaos. Therefore, based on the existing cooperative behavior of remanufacturing supply chain and the existing theoretical research, this paper mainly involves the following three points. First, the optimal government subsidy under supply chain cooperation and non-cooperative behavior is explored. Second, we analyze the dynamic effects under different supply chain behaviors. Lastly, the dynamic effects of cooperative behavior on multi-cycle decisions and profits are investigated.

The remainder of this paper is structured as follows. In section 2, we review the relevant literature. Section 3 presents the research background of this study through the model description and assumptions, and establishes the bounded rational dynamic decision model under cooperative and non-cooperative behavior. In Section 4, we perform numerical simulations of dynamic decisions, performance, optimal subsidy and possible complex system phenomena under cooperative behavior. In Section 5, we discuss the results. The final section provides directions for future research.

## 2. Literature review

We conducted a literature review from three aspects related to this study, i.e., authorized remanufacturing supply chain, cooperative behavior and government subsidies.

The entry of third-party remanufacturing into the market leads to fierce competition in the remanufactured product market. The original manufacturer balances the new product remanufacturing design, remanufacturing demand, and production inventory planning, which is more complex [19]. Third-party remanufacturing affects the market product demand of the original supply chain, and indirectly affects the operation of the original supply chain. Therefore, to develop the remanufacturer, many manufacturers aim to absorb the profits of the remanufacturing market through authorized remanufacturing [20]. However, the original manufacturer indirectly increases the competition because of authorization fees [21]. Wang et al. [13] argued that in the output decision of the original manufacturer and the third-party remanufacturer, the intrusion of the third-party remanufacturer would lead to the decline of the profit of the original manufacturer. Agrawal et al. [22] studied from the perspective of consumers’ perception of new products and believed that third-party remanufacturing might improve consumers’ perception of new products and thus increase the profit of the original manufacturer. To reduce the effect of remanufactured products on the original manufacturer’s profit, the original manufacturer can adopt methods, such as market segmentation [14, 23], product quality improvement [24, 25], and price discrimination [26, 27]. The above research provides the theoretical basis for the efficient operation of remanufacturing closed-loop supply chain from three perspectives: the pricing of remanufacturing and new products, the influence of remanufacturing on the profit of the new product supply chain and the influence of remanufacturing production mode on the original supply chain. And provide a theoretical basis for our study to examine the establishment of the original manufacturer and authorized third-party remanufacturing market models in monopoly markets. However, the aforementioned research work does not take into consideration the influence of a new product supply chain on an authorized remanufacturing supply chain through cooperative decision.

The cooperative behavior in this paper is the behavior of the centralized decision-making between the upstream and downstream in the supply chain [28–30]. Among the behaviors of supply chain participants, cooperative behavior has been widely studied because it can reduce information asymmetry in supply chain [31–33]. The supply chain cooperative decision can reduce the probability of supply chain interruption, increase market share, and improve performance [34–36]. According to the study in [37], a cooperative supply chain could achieve a higher social responsibility effect because it reduced internal competition. Hu et al. [38] found that the profits and environmental benefits of a cooperation model were higher than those of a non-cooperation model. On the other hand, the existing market decisions often deviate from optimal numerical decisions. And easy to generate inventory costs and sales losses. According to the study in [39], although most efforts to mitigate the bullwhip effect emphasized the process of information sharing, collaboration and coordination, few considered the cost of the supply chain affected by decision makers’ cognitive forms and behaviors. The study in [40] suggested that managers’ emotional responses in the face of major events or emergencies, such as the Coronavirus (COVID-19) pandemic, affect managers’ cognition, preventing them from recognizing the full scope of issues they face and updating their decision-making models accordingly. Based on the above studies on supply chain cooperative decision-making and influence of decision makers’ behavior, we found that few existing studies consider whether cooperative decision making is conducive to reducing the adverse influence of deviation from the optimal order when faced with large disturbances.

At present, the effects of government subsidies on enterprise decision-making are mainly studied by two methods: empirical research and mathematical modeling. In the empirical research method, the possible economic impact of government subsidy policies mainly includes the effect of government subsidy on enterprise innovation [41–43], enterprise anti-risk ability, enterprise performance, industrial chain efficiency [44–46], etc. Shi et al. [47] studied the impact of government subsidies on the labor productivity of enterprises under the upstream and downstream market forces of strategic emerging industries. The research shows that different degrees of market forces in the supply chain affect the selection mechanism of government subsidies. Xue and Feng [48] argued that government subsidies need to choose the right time to maximize the efficiency of subsidies. Zhang and Lin [49] found that market competition affects the effect of government subsidies on the total factor productivity of new energy enterprises. The above empirical research provides a rich theoretical basis for the effect of government subsidy policies. However, they do not consider the effect of government subsidies on market decision-making. And less consideration is given to the impact of government subsidies on chain-to-chain competition in remanufacturing supply chains. Through mathematical modeling and system simulation, the influence of government subsidies on remanufacturing supply chain market decision-making can be predicted.

The research on the effect of government subsidies on the remanufacturing supply chain through mathematical modeling mainly focuses on the effect of government subsidies in different situations, such as remanufacturing channels, remanufacturing modes and subsidy objects, etc. The metrics of the effects of government subsidies are profit [50, 51], environmental impact [52, 53], and social welfare [54–56]. In [57], appropriate government subsidies were shown to promote demand for green products and increase profits of the supply chain. Centralized supply chain profit was greater than decentralized supply chain profit. Chen et al. [58] found that under the government utility orientation, i.e., when the government considered both social welfare and subsidy costs, the profits of a centralized supply chain were lower than those of a decentralized supply chain, and the subsidy rate decreased with an increase in consumers’ preferences. Chai et al. [59] analyzed the effects of government subsidy policies and carbon cap-and-trade policies on remanufacturing in a closed-loop supply chain consisting of manufacturers, independent remanufacturers and retailers. The research shows that the government subsidy object is different, but the effect of promoting the development of remanufacturing is the same. The technology license fee is not conducive to the development of remanufacturing. Qiao and Su [51] studied the influence of government subsidies on variables such as profit, consumer surplus and social surplus in different remanufacturing competitive markets of closed-loop supply chains with game theory. The results suggest that the environmental impact of government subsidies is influenced by the relative carbon emissions of new and remanufactured products, as well as the competitive market environment of remanufacturing in the market. The above research not only provides theoretical basis but also provides new ideas for research. Based on the above research, we found that government subsidies might affect the profit advantage caused by cooperative decision-making. And, the above studies losed sight of the comparative study of optimal subsidies under cooperation behavior.

From the perspective of research methods, these studies were based on the assumption of complete rationality. Nonlinear interactions and feedback loops exist in economic systems. Additionally, the nonlinear dynamics theory based on bounded rationality has been widely applied in supply chain [60–62]. With the development of the supply chain and the entry of science and technology, factors affecting the decision-making stability of the supply chain increased, and studies on chaotic characteristics of the supply chain begin to increase [63–65]. Factors affecting supply chain stability include service value [66, 67], channel problem [68, 69], consumer preference [70], green investment level [71], etc. The research on the complexity and dynamics of closed-loop supply chain decision-making mainly considers the coupling dynamics between product sales and recovery in a closed-loop supply chain composed of manufacturers and retailers. Li et al. [72] established the stability of the reverse supply chain recycling competition model considering the behavior factors of recyclers. Zhan et al. [73] studied the complexity and dynamics of decision-making in a closed-loop supply chain composed of manufacturers with a recycling business but without direct sales channel and manufacturers without recycling business but with a direct sales channel under carbon tax. The research results showed that the system’s stability decreased with the increase of carbon tax. Zhang et al. [74] studied a two-channel closed-loop supply chain where, in the presence of carbon tax regulations, manufacturers sell new products through traditional channels through a fair care retailer and distribute remanufactured products through their direct channels. The high value of price adjustment speed, carbon tax rate or fair attention of retailers has a strong destabilizing effect on system stability.

However, studies on the dynamic influence of cooperative behavior under government policies on supply chain decisions are lacking. Therefore, based on the aforementioned studies, we analyze the complexity and dynamics of the impact of cooperative and non-cooperative behavior between retailers and manufacturers on government subsidies and market. Taking the development of government-subsidized remanufactured products and carbon tax as the background, the interactive effects of multi-cycle decisions of new and remanufactured products is studied. To the best of our knowledge, there has not been another research study that combines cooperative behavior, government subsidies, systematic multi-cycle decision-making, and performance analysis. The above analysis has a particular guiding significance for balancing the development of new and remanufactured products. And it provides reference for improving government subsidy mechanism for different supply chain situations.

## 3. Proposed model

In this section, we establish two supply chains whose decisions affect each other, i.e., the supply chain for new products and the supply chain for remanufacturing products. There are two types of behavior between retailers and manufacturers in the new product supply chain, i.e., cooperative and non-cooperative. Independent remanufacturers pay authorization fees to new product manufacturers. Low-cost remanufacturers prefer direct sales channels when authorization fees exist [55]. Therefore, remanufacturers with direct sales channels constitute remanufactured-product supply chains. To reduce carbon emissions and promote the development of remanufacturing, the government subsidizes remanufacturers.

Based on the cooperative behavior of retailers and manufacturers, we establish the following two decision models.

The first decision system consisted of an authorized remanufacturing supply chain and a non-cooperative new product supply chain (denoted as model N). In this model, manufacturers and retailers in the new product supply chain made the wholesale price and inventory decisions separately.

The second decision system consisted of an authorized remanufacturing supply chain and a cooperative new product supply chain (denoted as model C). In this mode, retailers and manufacturers form alliances to make decisions.

**Fig 1:**
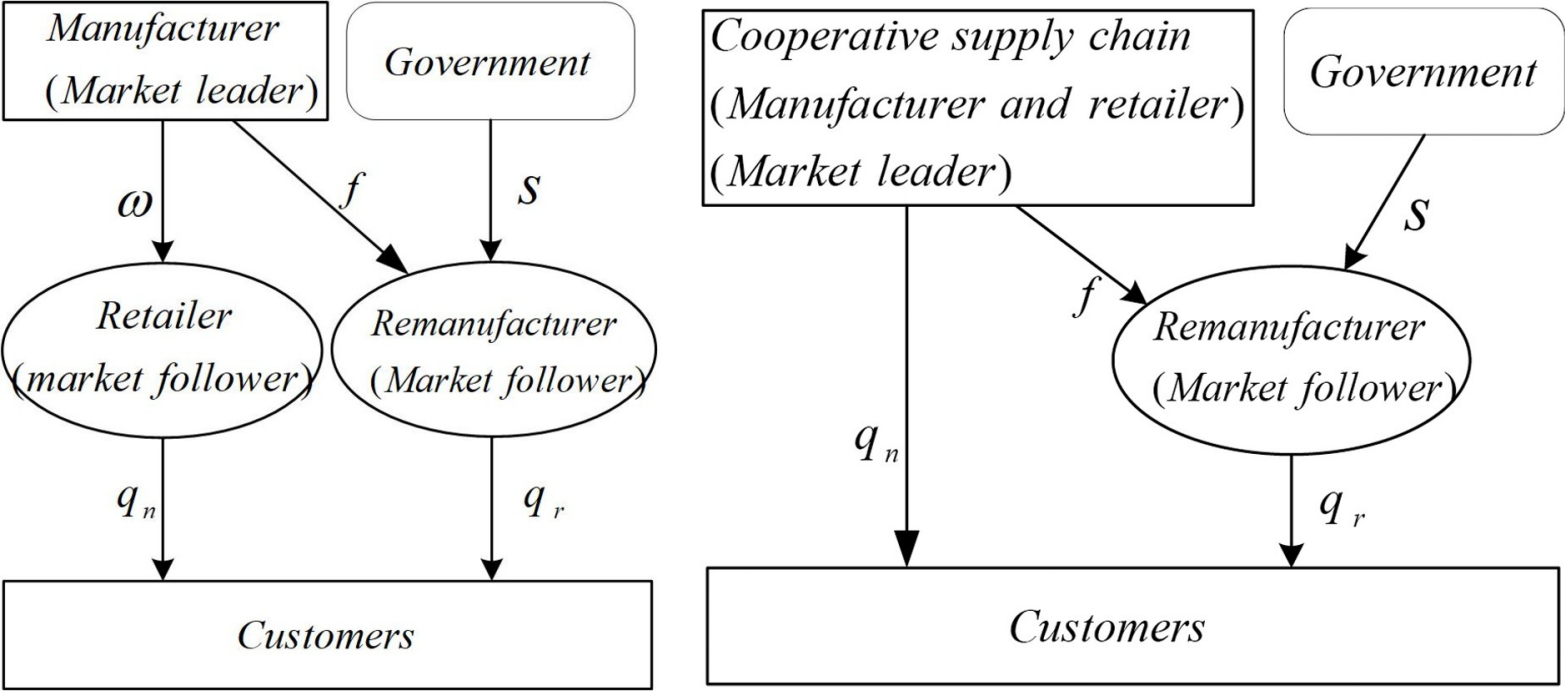
Structure of decision system. (a) Model N, and (b) Model C.

Fig 1 is the decision structure of supply chain. Table 1 shows the parameters and their meanings.

**Table 1:** Notations.

*δ*(0<*δ*<1)	Value discount of remanufactured products relative to new products
*c*	The unit production cost of new products
*e*	The unit new product’s carbon emission
*p*_*t*_	Carbon tax per unit product
*ε*	Cost savings per unit of remanufactured product
*k*(0<*k*<1)	Carbon emissions discounts for remanufactured products
*s*	Government subsidies
$q_{n}^{i}$	Market demand for new products in model *i*, *i*∈{*N*,*C*}
$q_{r}^{i}$	Market demand for remanufactured products in model *i*, *i*∈{*N*,*C*}
*f* ^*i*^	Unit remanufactured product authorization fee in model *i*, *i*∈{*N*,*C*}
*ω* ^*i*^	Unit new product wholesale price in model *i*, *i*∈{*N*,*C*}
*p*_*n*_	The new product price
*p*_*r*_	The remanufactured product price
$\prod_{m}^{N}$	The manufacturers’ profit in model *N*.
$\prod_{n}^{N}$	The retailer’s profit in model *N*.
$\prod_{r}^{i}$	The remanufacturer’s profit in model *i*, *i*∈{*N*,*C*}.
$\prod_{new}^{i}$	The new product supply chain’s profit in model *i*, *i*∈{*N*,*C*}.
*SW*_*i*_	Social welfare in model *i*, *i*∈{*N*,*C*}.

### 3.1. Model assumptions and construction

Based on the research objective, certain key assumptions are made in this paper that are described as follows.

*Assumption 1*. New product manufacturers are supply chain leaders [74]. A remanufactured product have the same functionality as a new product [13]. Remanufactured products cost less than new products [75].

*Assumption 2*. The manufacturer’s inventory is equal to the market demand. We do not consider residual product value or loss of product out of stock; this is the same in the studies in [15, 76].

*Assumption 3*. Supply chain members have a bounded rationality. In multi-period decision-making, the decider play the game during each decision-making period [77].

*Assumption 4*. The willingness of consumers to pay for the product is *υ*; *υ* follows a uniform distribution on the interval [0,1]. The market size is assumed to be 1. To ensure the existence of both the new and remanufactured products, the parameter *δ* have to be met, i.e., $\frac{p_{r}}{p_{n}}<\delta <1-p_{n}+p_{r}$.

Based on these assumptions, we build an inverse demand model for new and remanufactured products expressed as follows:

$$\{\begin{array}{l} p_{n}(q_{n},q_{r})=1-q_{n}-\delta q_{r}\\ p_{r}(q_{n},q_{r})=\delta (1-q_{n}-q_{r}) \end{array}$$ (1)

In Eq (1), *δ* represents the value discount of remanufactured products relative to that of new products; the market size is normalized to 1. *δ* = 1 means that consumers think that new and remanufactured products have the same value. The inverse demand model has been widely used [13, 15]. The detailed derivation of the inverse demand model is presented in Appendix A in S1 File.

According to Eq (1), we obtain the new product manufacturer’s profit expressed as follows:

$$\prod_{m}(\omega ,f)=(\omega -c-ep_{t})q_{n}+fq_{r}$$ (2)

In Eq (2), the new product manufacturer’s profit consists of the profit from selling the new product to retailers and the authorization fees paid by the third-party remanufacturer.

The retailer’s profit is expressed as

$$\prod_{n}(q_{n})=(p_{n}-\omega )q_{n}$$ (3)

The remanufacturer’s profit is expressed as

$$\prod_{r}(q_{r})=(p_{r}-c-kep_{t}+\varepsilon )q_{r}-fq_{r}+sq_{r}$$ (4)

In Eq (3), the retailer’s profit consists of only the retailer’s sales profit. In Eq (4), the remanufacturer’s profit consists of the direct sales channel’s product profit, the authorization fee and government subsidies.

Considering the impact of remanufactured products on the new product market, the original manufacturer intends to control the product prices through wholesale prices. To reduce the impact of remanufactured products on new product sales, retailers manage the inventory of new products. New product retailers carry out product differentiation strategies and do not compete with remanufacturers in terms of price. Therefore, product inventory is the decision variable for the remanufacturer and retailer.

The model applies to products with high brand image and quality. The original manufacturer can segment the market through price and obtain remanufacturing profits using brand value to authorize a third party. The original manufacturer’s production reduction do not affect the unit cost of the product.

In the following two sections, we separately build and solve the decision model for different behaviors.

### 3.2. Model N

In Model N, the new product manufacturer is the market leader. Retailers and remanufacturers are market followers who decide on inventory based on the manufacturer’s decision. There is no cooperation in the supply chain. The decision structure is given by Eq (5):

$$\left\{ \begin{array}{l} \underset{\omega ,f}{Max}\prod_{m}^{N}\\ s.t.\left\{ \begin{array}{c} q_{n}^{N}=\arg\;\underset{q_{n}}{Max}\prod_{n}^{N}\\ q_{r}^{N}=\arg\;\underset{q_{r}}{Max}\prod_{r}^{N} \end{array}\right. \end{array}\right.$$ (5)

We solve Eq (5) by backward induction and obatin the decision-maker’s marginal profit using Eq (6):

$$\{\begin{array}{l} \frac{\partial \prod_{m}^{N}}{\partial \omega }=\frac{-2-3c-2f-2ep_{t}-kep_{t}+s+4\omega +\delta +\varepsilon }{\delta -4}\\ \frac{\partial \prod_{m}^{N}}{\partial f}=\frac{4f-2s-\delta -2\omega \delta +c(2+\delta )+ep_{t}(2k+\delta )-2\varepsilon }{(\delta -4)\delta }\\ \frac{\partial \prod_{n}^{N}}{\partial q_{n}}=1-2q_{n}-\omega -q_{r}\delta \\ \frac{\partial \prod_{r}^{N}}{\partial q_{r}}=-c-f-kep_{t}+s+\delta (1-q_{n}-2q_{r})+\varepsilon \end{array}$$ (6)

Supply chain participants may not be able to obtain all information. Additionally, decisions are made based on bounded rational expectations. The decision making process is complex and periodic. Therefore, this study makes decisions based on marginal profits. The decision function is expressed as follows:

$$\left\{ \begin{array}{l} \omega_{t+1}^{N}=\omega_{t}^{N}+\alpha_{1}\omega_{t}^{N}(\frac{-2-3c-2f_{t}^{N}-2ep_{t}-kep_{t}+s+4\omega_{t}^{N}+\delta +\varepsilon }{\delta -4})\\ f_{t+1}^{N}=f_{t}^{N}+\alpha_{2}f_{t}^{N}(\frac{4f_{t}^{N}-2s-\delta -2\omega_{t}^{N}\delta +c(2+\delta )+ep_{t}(2k+\delta )-2\varepsilon }{(\delta -4)\delta })\\ q_{n,t+1}^{N}=q_{n,t}^{N}+\alpha_{3}q_{n,t}^{N}(1-2q_{n,t}^{N}-\omega_{t+1}^{N}-q_{r,t}^{N}\delta )\\ q_{r,t+1}^{N}=q_{r,t}^{N}+\alpha_{4}q_{r,t}^{N}(-c-f_{t+1}^{N}-kep_{t}+s+\delta (1-q_{n,t}^{N}-2q_{r,t}^{N})+\varepsilon ) \end{array}\right.$$ (7)

By solving for the stable decision value of the decision system in Eq (7), we obtain an economically meaningful equilibrium solution for the decision system as follows:

$$\{\begin{array}{l} \omega^{N}=\frac{1}{2}(1+c+p_{t}e)\\ f^{N}=\frac{1}{2}(s+\delta +\varepsilon -c-kep_{t})\\ q_{n}^{N}=\frac{s+\delta +\varepsilon +c-2-e(k-2)p_{t}}{2(\delta -4)}\\ q_{r}^{N}=\frac{2(c+kep_{t}-s-\varepsilon )-\delta (1+c+ep_{t})}{2\delta (\delta -4)} \end{array}$$ (8)

The Jacobian matrix of the above equilibrium solution in the system is expressed as follows:

$$J^{N}=[\begin{array}{cccc} \frac{-4+\delta +\alpha_{1}j_{1}}{\delta -4} & \frac{2\omega^{N}\alpha_{1}}{4-\delta } & 0 & 0\\ \frac{2f^{N}\alpha_{2}}{4-\delta } & \frac{\alpha_{2}j_{2}-4\delta +\delta^{2}-2\varepsilon }{\delta (\delta -4)} & 0 & 0\\ -q_{n}^{N}\alpha_{3} & 0 & 1-\alpha_{3}(\omega^{N}+4q_{n}^{N}+q_{r}^{N}\delta -1) & -q_{n}^{N}\alpha_{3}\delta \\ 0 & -q_{r}^{N}\alpha_{4} & -q_{r}^{N}\alpha_{4}\delta & 1-2q_{r}^{N}\alpha_{4}\delta -\alpha_{4}j_{3} \end{array}]$$ (9)

where

$$j_{1}=-2-3c-2f^{N}-2ep_{t}-kep_{t}+s+8\omega^{N}+\delta +\varepsilon$$

$$j_{2}=8f^{N}+2kep_{t}-2s-\delta +ep_{t}\delta -2\omega^{N}\delta +c(2+\delta )-2\varepsilon$$

$$j_{3}=c+f^{N}+kep_{t}-s-\delta +q_{n}^{N}\delta +2q_{r}^{N}\delta -\varepsilon$$

Then, we obtain the characteristic equation of the Jacobian matrix expressed as follows:

$$Y(\lambda )=\lambda^{4}+y_{1}\lambda^{3}+y_{2}\lambda^{2}+y_{3}\lambda +y_{4}$$ (10)

where *y*_*i*_(*i* = 1,2,3,4) is presented in Appendix B in S1 File.

According to Eq (10), through the Jury condition, we obtain the requirements to maintain the equilibrium point of the supply chain decision-making system, expressed as follows:

$$\left\{ \begin{array}{l} 1+y_{1}+y_{2}+y_{3}+y_{4}>0\\ 1-y_{1}+y_{2}-y_{3}+y_{4}>0\\ 1-|y_{4}|>0\\ |1-y_{4}{}^{2}|-|y_{3}-y_{1}y_{4}|>0\\ |{\left(1-y_{4}{}^{2}\right)}^{2}-{\left(y_{3}-y_{1}y_{4}\right)}^{2}|-|\left(1-y_{4}^{2})(y_{1}-y_{4}y_{2})-(y_{4}y_{3}-y_{1})(y_{4}y_{1}-y_{3}\right)|>0 \end{array}\right.$$ (11)

According to Eq (11), we obtain a reasonable range of parameters to maintain system stability. Because there are many parameters and the stable conditions are complex and can not be expressed mathematically, we analyze them through computer simulations in Section 4.

### 3.3. Model C

In Model C, the manufacturer and retailers cooperate to determine the optimal product inventory. The decision structure is expressed as follows:

$$\left\{ \begin{array}{l} \underset{q_{n},f}{Max}\prod_{new}^{c}\\ \;s.t.q_{r}^{c}=\arg\;\underset{q_{r}}{Max}\prod_{r}^{c} \end{array}\right.$$ (12)

Where $\prod_{new}^{c}(q_{n,}f)=fq_{r}-q_{n}^{2}-q_{n}(c-1+ep_{t}+\delta q_{r})$

We solve Eq (12) by backward induction and obtain the decision-maker’s marginal profit using Eq (13):

$$\{\begin{array}{l} \frac{\partial \prod_{new}^{c}}{\partial f}=\frac{-c-2f-kep_{t}+s+\delta +\varepsilon }{2\delta }\\ \frac{\partial \prod_{new}^{c}}{\partial q_{n}}=\frac{1}{2}(2-c+e(k-2)p_{t}-4q_{n}-s-\delta +2q_{n}\delta -\varepsilon )\\ \frac{\partial \prod_{r}^{c}}{\partial q_{r}}=-c-f-kep_{t}+s+\delta (1-q_{n}-2q_{r})+\varepsilon \end{array}$$ (13)

Similar to Model N in Section 3.2, we establish the following nonlinear decision system:

$$\{\begin{array}{l} f_{t+1}^{c}=f_{t}^{c}+\alpha_{2}f_{t}^{c}(\frac{-c-2f_{t}^{c}-kep_{t}+s+\delta +\varepsilon }{2\delta })\\ q_{n,t+1}^{c}=q_{n,t}^{c}+\alpha_{3}q_{n,t}^{c}(\frac{1}{2}(2-c+e(k-2)p_{t}-4q_{n,t}^{c}-s-\delta +2q_{n,t}^{c}\delta -\varepsilon ))\\ q_{r,t+1}^{c}=q_{r,t}^{c}+\alpha_{4}q_{r,t}^{c}(-c-f_{t+1}^{c}-kep_{t}+s+\delta (1-q_{n,t+1}^{c}-2q_{r,t}^{c})+\varepsilon ) \end{array}$$ (14)

By solving the above nonlinear decision system, we obtain an economically meaningful equilibrium solution for the supply chain decision system, expressed as follows:

$$\{\begin{array}{l} f^{c}=\frac{1}{2}(s-c-kep_{t}+\delta +\varepsilon )\\ q_{n}^{c}=\frac{c-2-e(k-2)p_{t}+s+\delta +\varepsilon }{2(\delta -2)}\\ q_{r}^{c}=\frac{s+c(\delta -1)+ep_{t}(\delta -k)+\varepsilon }{2(2-\delta )\delta } \end{array}$$ (15)

The Jacobian matrix in Eq (15) in the system is expressed as follows:

$$J^{c}=[\begin{array}{ccc} \frac{c\alpha_{2}+kep_{t}\alpha_{2}-s\alpha_{2}+2\delta -\alpha_{2}\delta -\alpha_{2}\varepsilon }{2\delta } & 0 & 0\\ 0 & 1+\frac{\alpha_{3}(c-e(k-2)p_{t}+s+\delta +\varepsilon -2)}{2} & 0\\ \frac{\alpha_{4}(s+c(\delta -1)+ep_{t}(\delta -k)+\varepsilon )}{2(\delta -2)\delta } & \frac{\alpha_{4}(s+c(\delta -1)+ep_{t}(\delta -k)+\varepsilon )}{2(\delta -2)} & \frac{-2+s\alpha_{4}+c\alpha_{4}(\delta -1)+\delta +ep_{t}\alpha_{4}(\delta -k)+\alpha_{4}\varepsilon }{\delta -2} \end{array}]$$ (16)

The characteristic equation of the Jacobian matrix in Eq (16) is expressed as follows:

$$U(\lambda )=\lambda^{3}+u_{1}\lambda^{2}+u_{2}\lambda +u_{3}$$ (17)

where *u*_*i*_(*i* = 1,2,3) is presented in Appendix C in S1 File.

The following conditions have to be satisfied for the equilibrium point to remain stable in the system.

$$\{\begin{array}{l} 1+u_{1}+u_{2}+u_{3}>0\\ 1-u_{1}+u_{2}-u_{3}>0\\ 1-|u_{3}|>0\\ |1-u_{3}{}^{2}|-|u_{2}-u_{1}u_{3}|>0 \end{array}$$ (18)

The characteristic polynomial coefficients can be used to evaluate the possible state of the system with a change in the decision adjustment parameters. Next, we compare the decision-making situation of the supply chain under cooperative and non-cooperative behaviors using numerical simulations.

## 4. Numerical simulations and analysis

Based on the nonlinear discrete system model in Section 3 and the deduced system stability conditions, we first study how cooperative behavior affects the effect of government subsidies aimed at maximizing social welfare. Then, we study the effect of cooperative behavior on multi-cycle decision-making. Finally, we examine the performance of multi-cycle decision-making.

The simulation parameters are as follows: *δ* = 0.7,*c* = 0.1,*e* = 0.1,*p*_*t*_ = 0.5,*ε* = 0.05,

*k* = 0.4,*α*_1_ = 1,*α*_2_ = 2,*α*_3_ = 2,*α*_4_ = 2.

### 4.1. Effect of government subsidies on system

Here, we analyze the impact of government subsidies from four aspects, i.e., systematic carbon emissions, consumer surplus, social welfare, remanufacturing profits and stable regions.

First, we calculate social welfare in cooperative and non-cooperative models expressed as follows:

$$\begin{array}{l} SW_{N}=\frac{1}{8{\left(-4+\delta \right)}^{2}\delta }(-4s^{2}+28\delta +8s\delta +3s^{2}\delta -9\delta^{2}+\delta^{3}+c^{2}(28+(-9+\delta )\delta )+e^{2}p_{t}{}^{2}(-2k\delta^{2}+k^{2}(-4+3\delta )+\delta (-4+3\delta ))\\ +24s\varepsilon +24\delta \varepsilon -2s\delta \varepsilon -4\delta^{2}\varepsilon +28\varepsilon^{2}-5\delta \varepsilon^{2}+2c\left(\begin{array}{l} ep_{t}(4\delta +k(12+(-9+\delta )\delta ))-4(3s+10\delta +7\varepsilon )\\ +\delta (-s(-9+\delta )+23\delta +21\varepsilon -3\delta (\delta +\varepsilon )) \end{array}\right)\\ -2ep_{t}(k(-4s+4\delta +3s\delta +12\varepsilon -\delta \varepsilon )+\delta (12-8\varepsilon +\delta (-9-s+\delta +\varepsilon )))) \end{array}$$ (19)

$$\begin{array}{l} SW_{C}=\frac{1}{8{\left(-2+\delta \right)}^{2}\delta }(-s^{2}+12\delta +s^{2}\delta -12\delta^{2}+3\delta^{3}+c^{2}(7-5\delta +\delta^{2})+e^{2}p_{t}{}^{2}(k^{2}(-1+\delta )-2k(-1+\delta )\delta +\delta (-4+3\delta ))\\ +6s\varepsilon -2s\delta \varepsilon +7\varepsilon^{2}-3\delta \varepsilon^{2}-2ep_{t}(ks(-1+\delta )+\delta (4+s-s\delta +\delta^{2}+\delta (-4+\varepsilon )-3\varepsilon )-k(-3+\delta )\varepsilon )\\ +2c(-12\delta +12\delta^{2}-3\delta^{3}-s(3-4\delta +\delta^{2})+ep_{t}(\delta +k(3-4\delta +\delta^{2}))-7\varepsilon +10\delta \varepsilon -3\delta^{2}\varepsilon )) \end{array}$$ (20)

**Fig 2:**
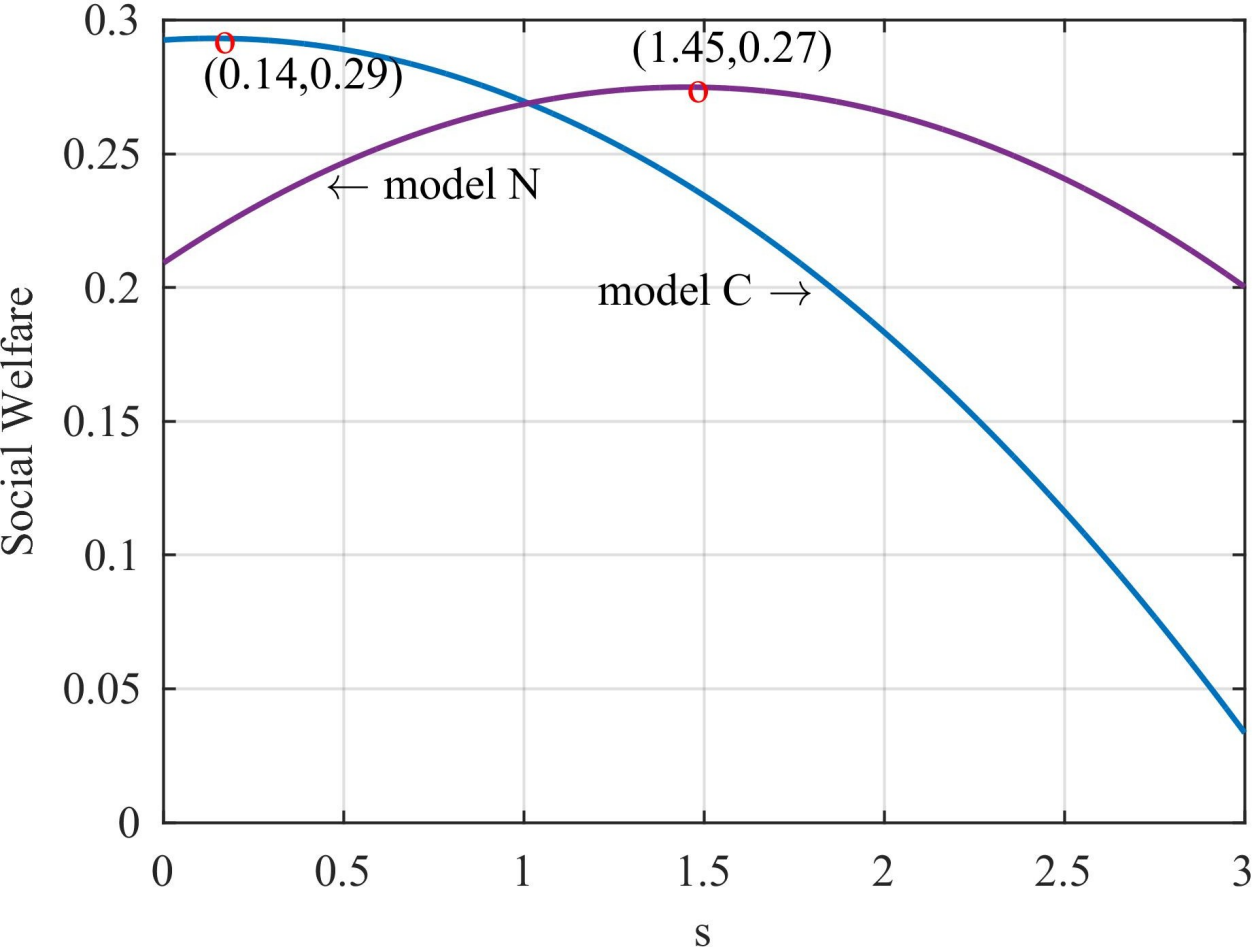
Social welfare variation with government subsidies.

The social welfare in Eqs (19) and (20) is a concave function of government subsidy, and there is an optimal social subsidy to maximize social welfare. According to social welfare, we can obtain the changes of social welfare with subsidies as shown in Fig 2. In comparison with the non-cooperative model, the cooperative model has a smaller optimal government subsidy (red circle in the blue line) than the non-cooperative model (red circle in the purple line).

To analyze the reasons for the above phenomenon, we analyze the carbon tax, consumer surplus and supply chain profits that affect social welfare.

**Fig 3:**
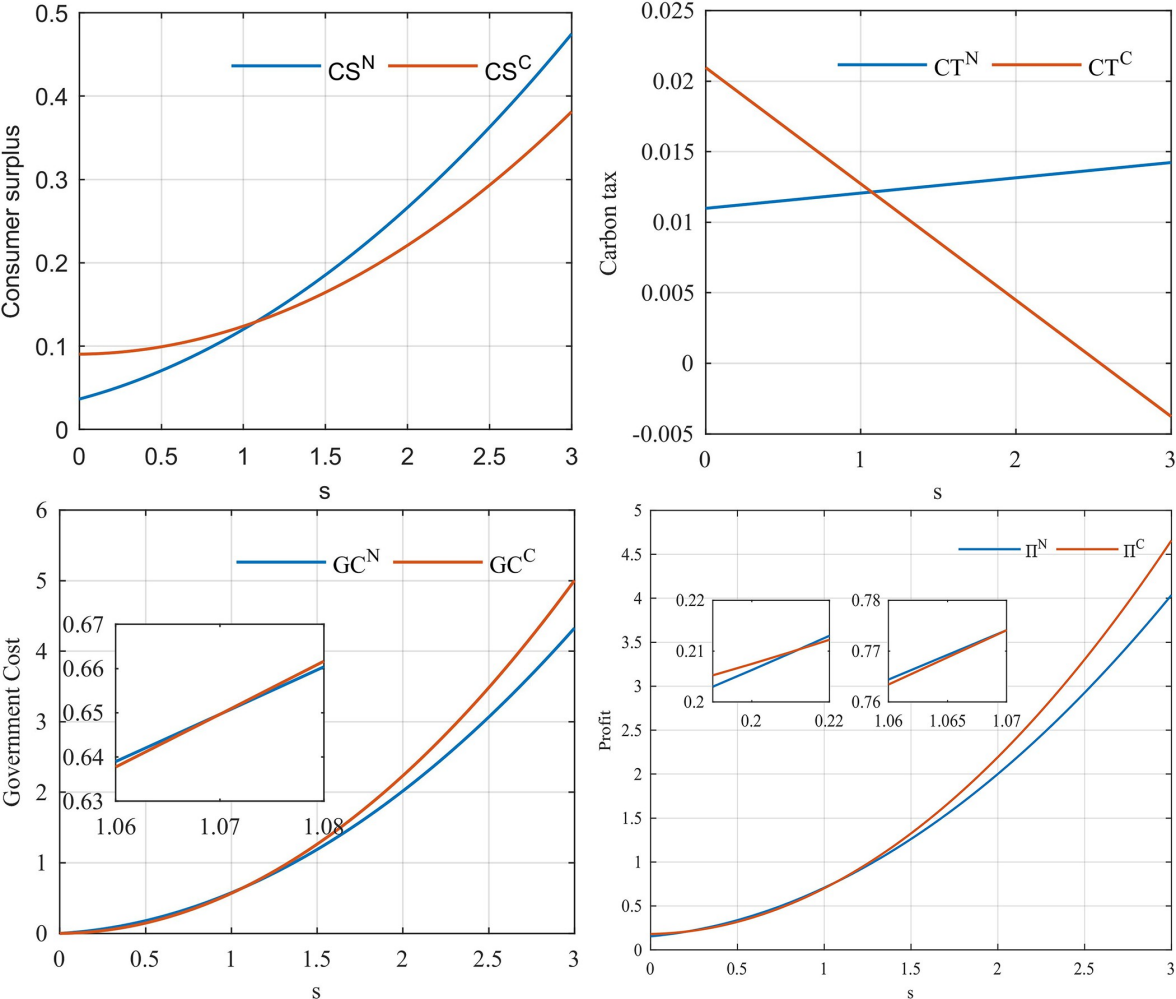
Impact of government subsidies. (a) The consumer surplus vary with the subsidy. (b) Carbon taxes vary with subsidies. (c) Effect of government subsidies on subsidy costs. (d) Effect of government subsidies on supply chain profits.

Fig 3(A) shows the change in consumer surplus with government subsidies. We can see that when there are no subsidies, the consumer surplus of cooperative supply chain is larger than that of non-cooperative supply chain. Fig 3(B) shows the government’s carbon tax with the increase of government subsidies. We can see that the direction of carbon tax revenue changes with subsidies is opposite under cooperation and non-cooperation. However, the gap in carbon tax revenues in the two models is small compared with the gap in consumer surplus in the two models when there are no subsidies. Fig 3(C) shows the change of the total cost of government subsidies with the increase of government subsidies. Although subsidy cost is a factor affecting the reduction of the optimal subsidy, it is not the main factor of the gap between cooperative and non-cooperative subsidies. Because when the subsidy size is above 1.07, the subsidy cost of non-cooperative supply chain is greater than that of cooperative supply chain. Fig 3(D) shows the change of profits with government subsidies. The initial supply chain profit gap between the two models is also very small. By comparing Fig 3(A) and 3(D), we find that consumer surplus, profits, and government subsidy costs have the same trend with subsidies, except for carbon taxes. However, the carbon tax revenue gap is small, whereas the consumer surplus initial gap is large in the two models. Therefore, we conclude that consumer surplus is the main factor affecting the subsidy gap between the two models.

**Fig 4:**
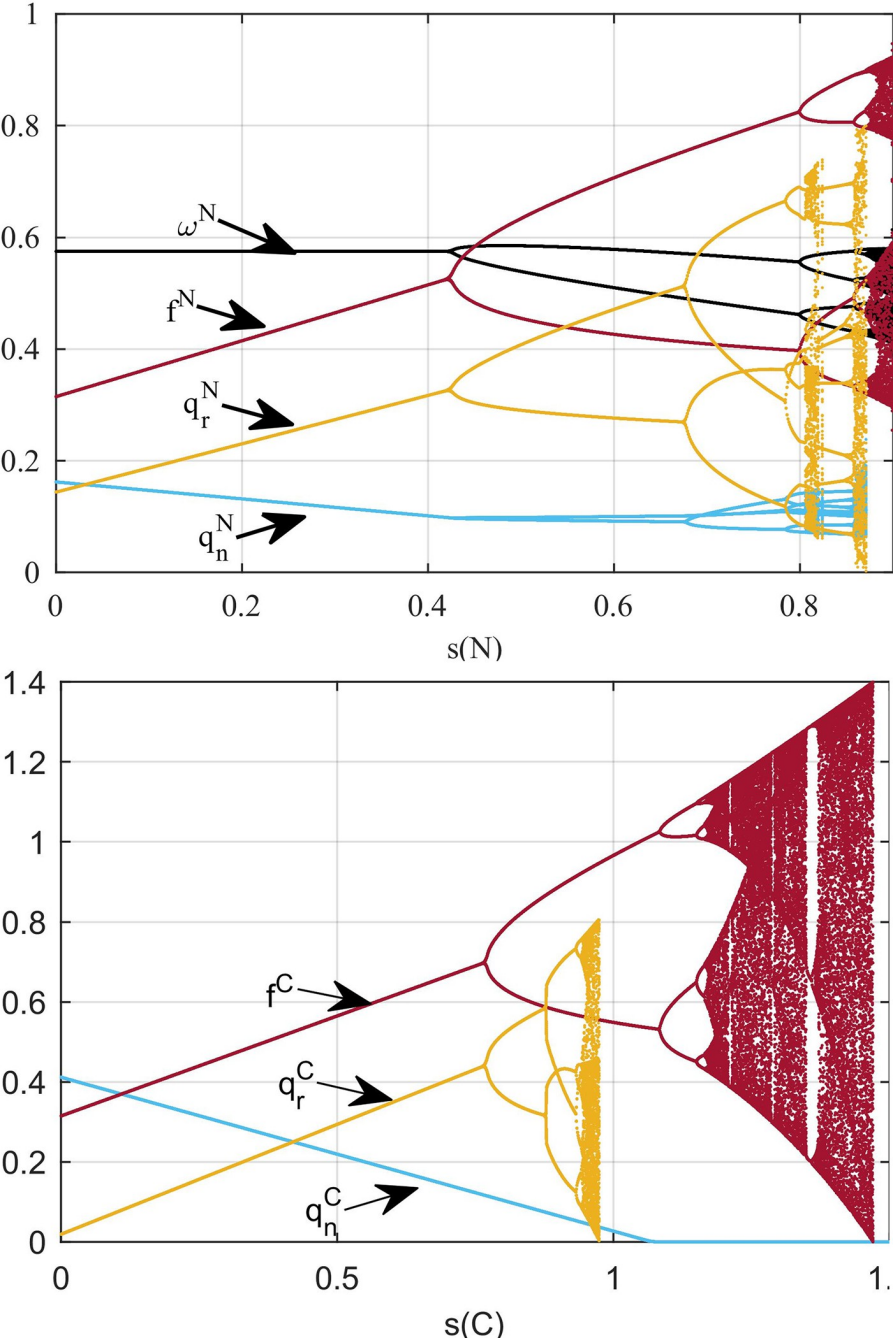
**Influence of government subsidies in multi-cycle decision-making.** (a) Model N, and (b) Model C.

Fig 4 shows the impact of government subsidies on system stability. From the comparison between Fig 4(A) and 4(B), we find that the stability of non-cooperative supply chain is more likely to be affected by government subsidies. In Fig 4(A), when the government subsidy is 0.19, the decision enters the bifurcation. In Fig 4(B), when the government subsidy is 0.78, the decision enters the bifurcation.

Through the combined analysis of Figs 2 and 4, we conclude that in the non-cooperative state, government subsidies aimed at maximizing social welfare lead to complex market decisions. However, when cooperative behavior exists in the supply chain, subsidies with the goal of maximizing social welfare will not affect the stability of the market decisions.

**Fig 5:**
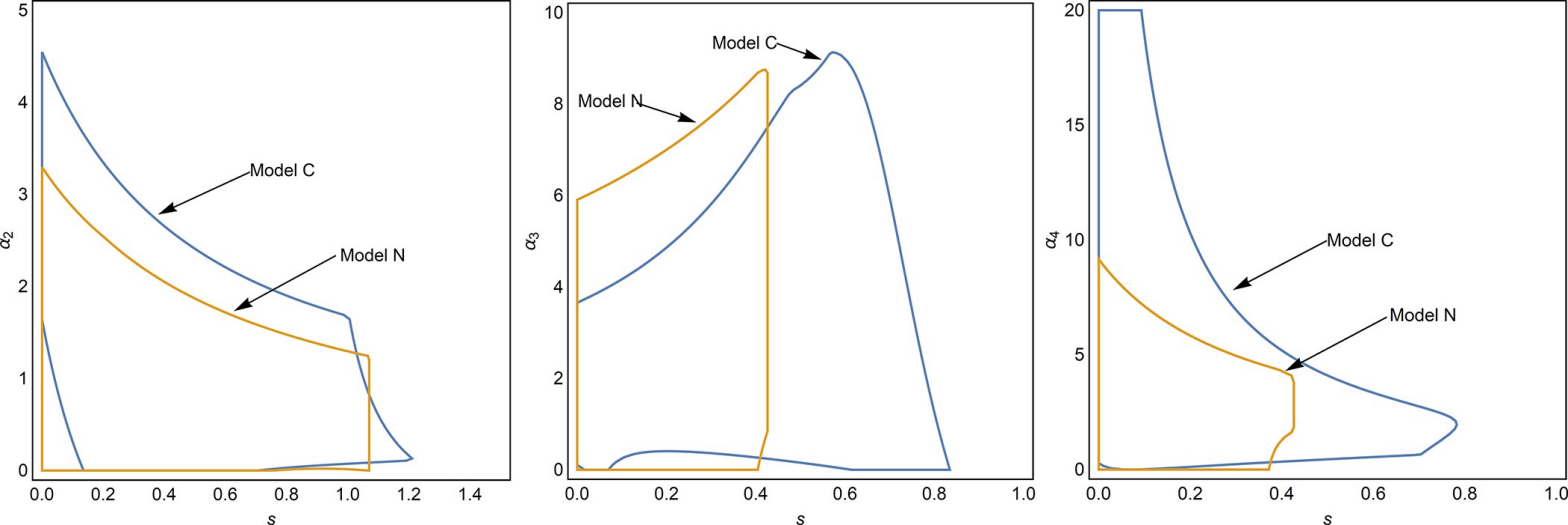
The decision adjustment parameters vary with subsidies. (a) Effect of *s* on *α*_2_. (b) Effect of *s* on *α*_3_. (c) Effect of *s* on *α*_4_.

Fig 5 shows the effect of government subsidy on the reasonable range of system decision adjustment parameters. In the two models, government subsidies have the same tendency to influence the reasonable range of decision parameters. Cooperative behavior is conducive to the stability of authorization costs and remanufacturing inventory decisions, but not conducive to the stability of new product inventory decisions.

Although the government subsidizes the authorization of remanufacturers, the stability range of decision adjustment parameters of remanufacturers is negatively correlated with government subsidies (Fig 5(C)). However, the stability range of the decision adjustment parameters of the retailers increases (Fig 5(B)). Additionally, in model C, the stability region of *α*_4_ is more sensitive to *s* in Fig 5(C).

### 4.2. Comparison of stability ranges under cooperation and non-cooperation

The stability of the system is affected not only by government subsidies but also by decision adjustment parameters. This section analyzes the dynamic evolution process of supply chain participant decision making in two models. We apply the bifurcation diagram, largest Lyapunov exponent (LLE), and attractor diagram to analyze the complexity caused by the change in decision adjustment parameters.

**Fig 6:**
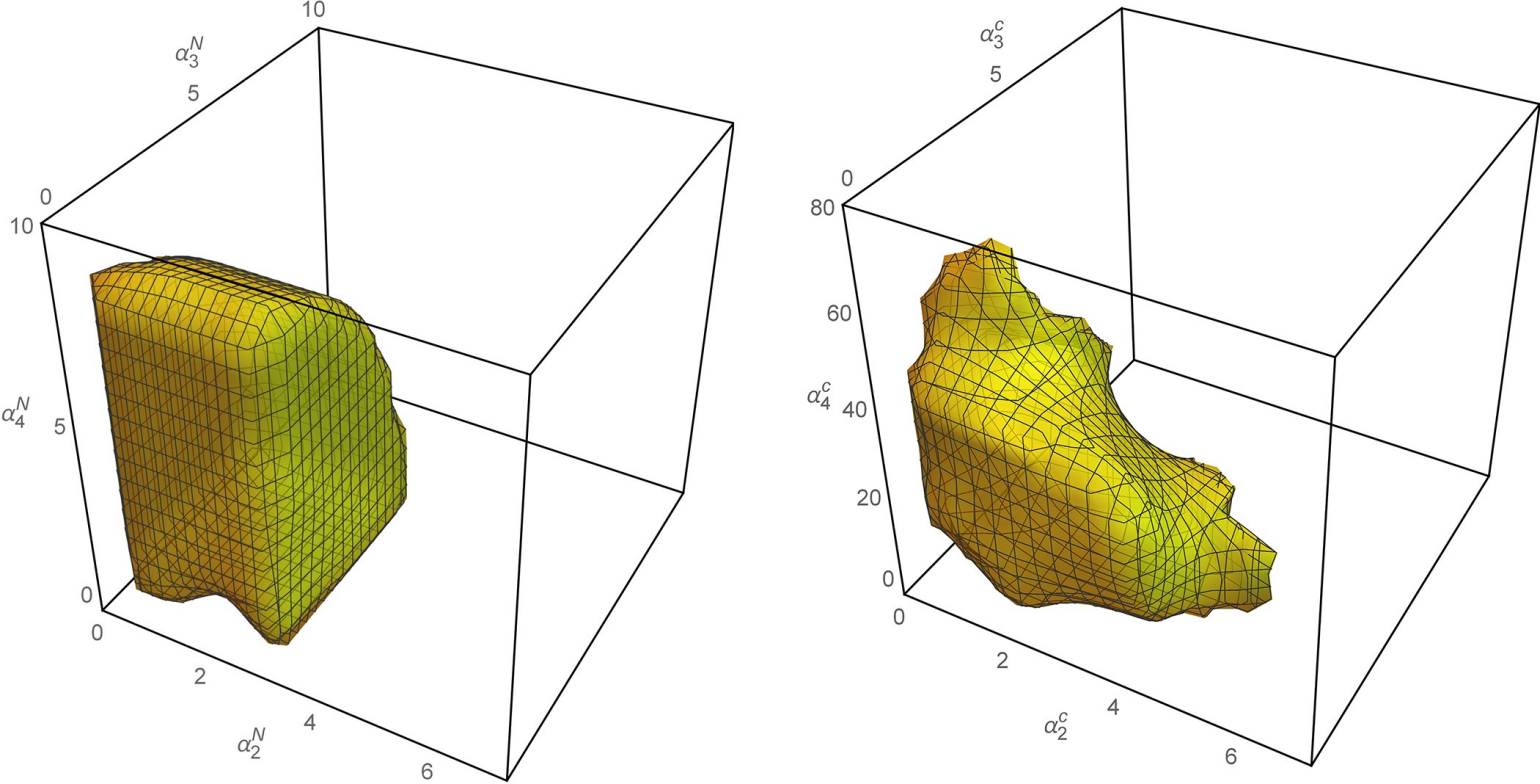
Three-dimensional stability range of system. (a) Model N, and (b) Model C.

We can obtain the interaction effect between *α*_2_, *α*_3_, and *α*_4_, as shown in Fig 6. To compare the three-dimensional (3-D) stable range of the system in Fig 6(A) and 6(B), we consider that the reasonable range of the system decision adjustment parameters in model C is more complex. The adjusted stability range of *α*_4_ increases significantly in Model C. The wholesale price of new products after supply chain cooperation is an internal variable that can not be compared with the wholesale price before cooperation. Therefore, we set *α*_1_ = 1 and do not consider its influence.

**Fig 7:**
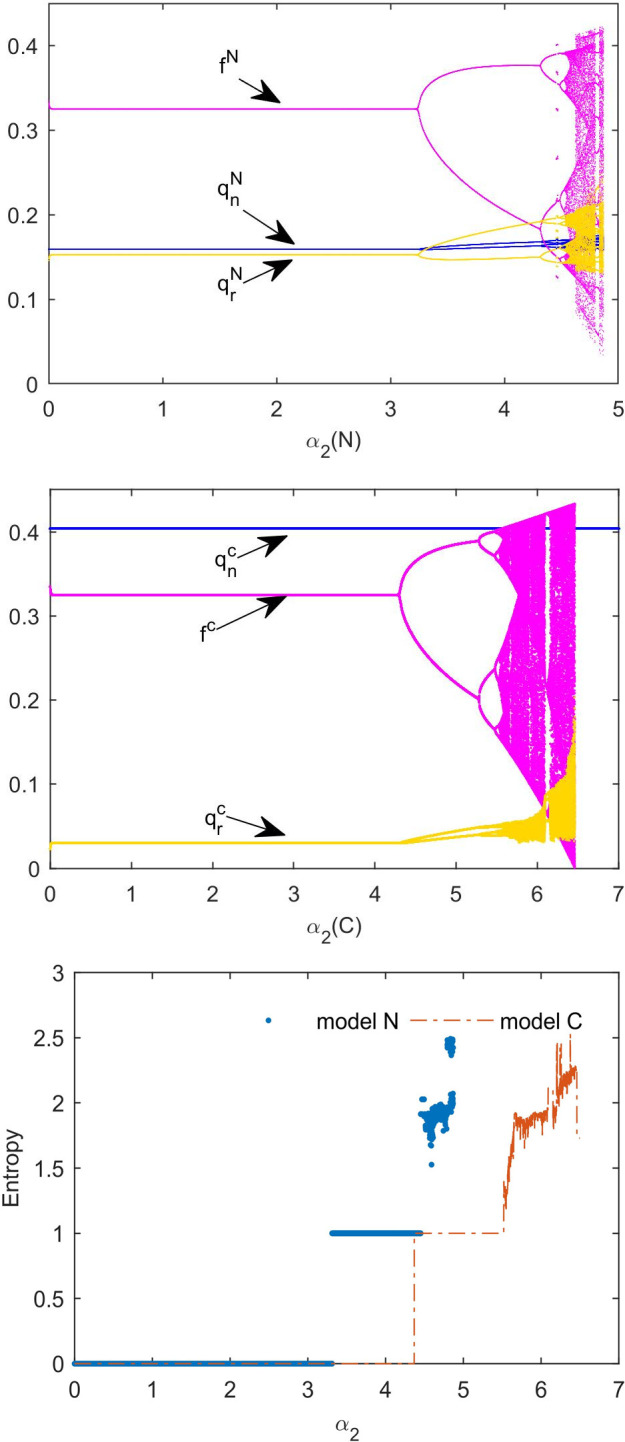
Influence of *α*_2_ on multi-cycle decision-making. (a) Bifurcation diagram with *α*_2_ in model N. (b) Bifurcation diagram with *α*_2_ in model C. (c) The system’s entropy with varying *α*_2_.

Fig 7 is the bifurcation diagram of the influence of *α*_2_ in models N and C. To compare Fig 7(A) and 7(B), we see that in model C, the inventory of new products is significantly increased, whereas the inventory of remanufactured products is decreased and the adjustment of the inventory of new products is not affected by the adjustment of authorization fees. The authorization fees remain the same in both models. However, the stability range of authorization fee adjustment parameters in model C is larger than that in model N. We can observe from Fig 7(C) that the entropy of the system in model C increases with varying *α*_2_ later than that in model N. Under the same adjustment parameters, non-cooperative supply chains may require more information to make authorization fee decisions.

**Fig 8:**
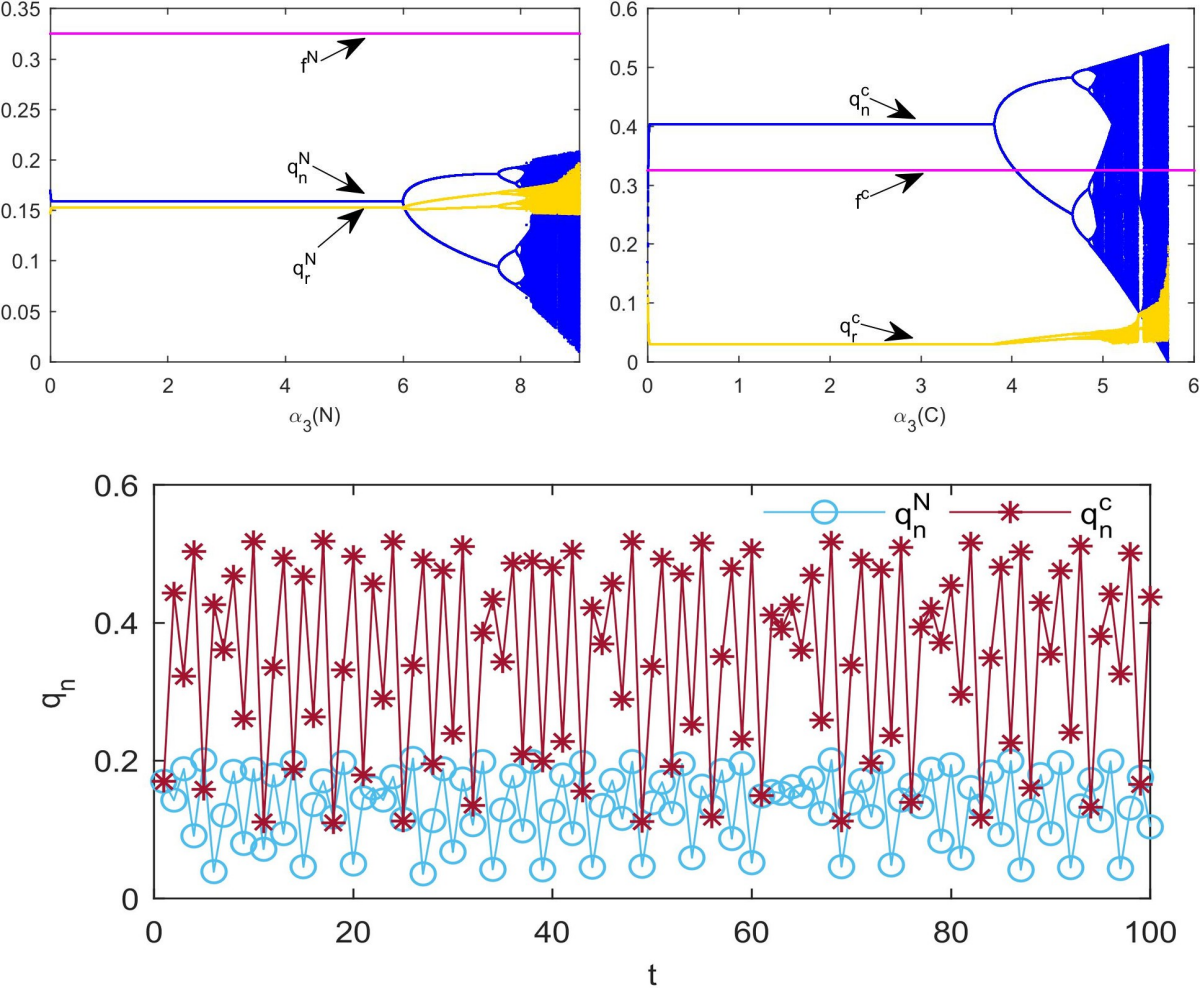
Influence of *α*_3_ on multi-cycle decision-making. (a) Bifurcation diagram with *α*_3_ in model N. (b) Bifurcation diagram with *α*_3_ in model C. (c) Time series of *q*_*n*_ with *α*_3_ = 8.7 in model N and *α*_3_ = 5.3 in model C.

Fig 8 shows the bifurcation diagram of the impact of *α*_3_ change on the system inventory in the two models. To compare Fig 8(A) and 8(B), we find that the stable range of *α*_3_ decreases after cooperation. From the fluctuation of the inventory in chaos, we can see that the fluctuation of new product inventory in model C (0~0.5) is larger than that in model N (0~0.2). Fig 8(C) shows the time series diagram of the inventory of new products under the condition of market turbulence. In comparison with the new product inventory in model N (blue line in Fig 8(C)), we can find that the new product inventory in model C fluctuates significantly (red line in Fig 8(C)).

**Fig 9:**
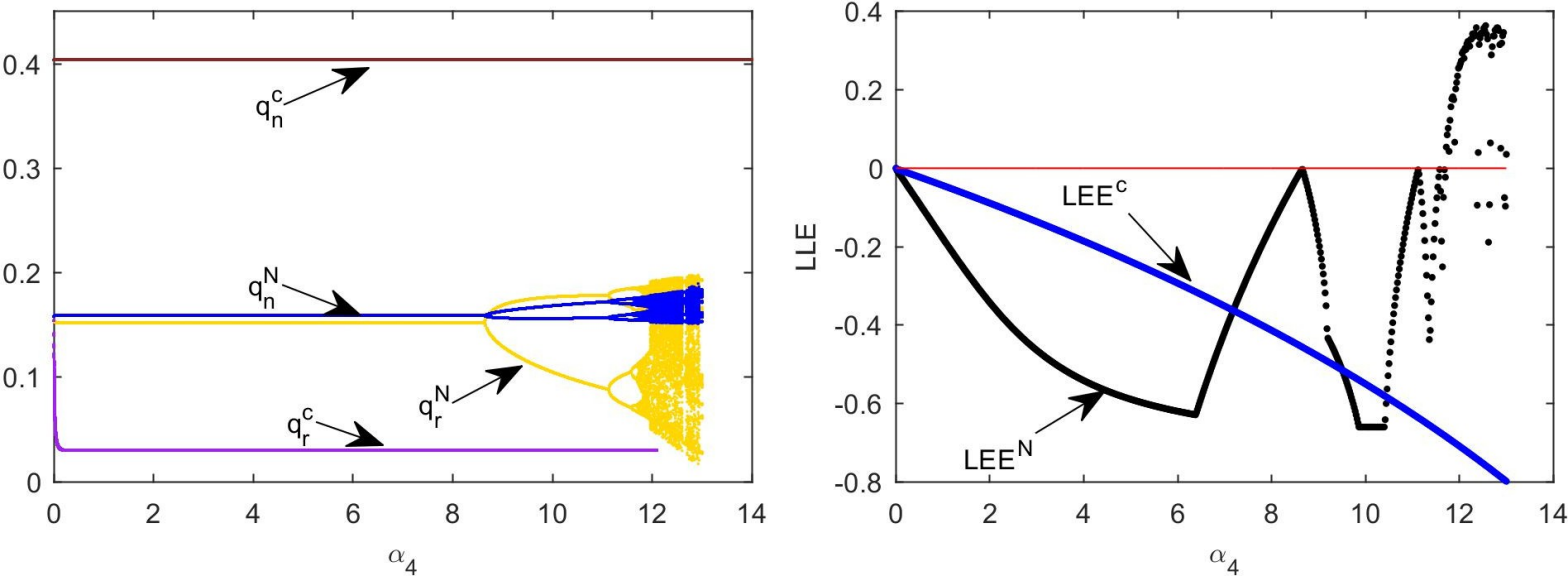
Influence of *α*_4_ on multi-cycle decision-making. (a) Bifurcation diagram with *α*_4_. (b) The largest Lyapunov exponent with *α*_4_.

Fig 9 shows the impact of *α*_4_ change on the inventory in the two models. By comparing Fig 9(A) and 9(B), we find that in model C, the inventory decision adjustment parameters of independent remanufacturers barely influence the system to enter chaos; however, when *α*_4_ exceeds a certain threshold, the decision value will enter the escape state. The LLE of the system in Fig 9(B) reflects two different system states as *α*_4_ changes. We can observe that the LLE in the cooperative decision system is always negative, which means the system is stable.

**Fig 10:**
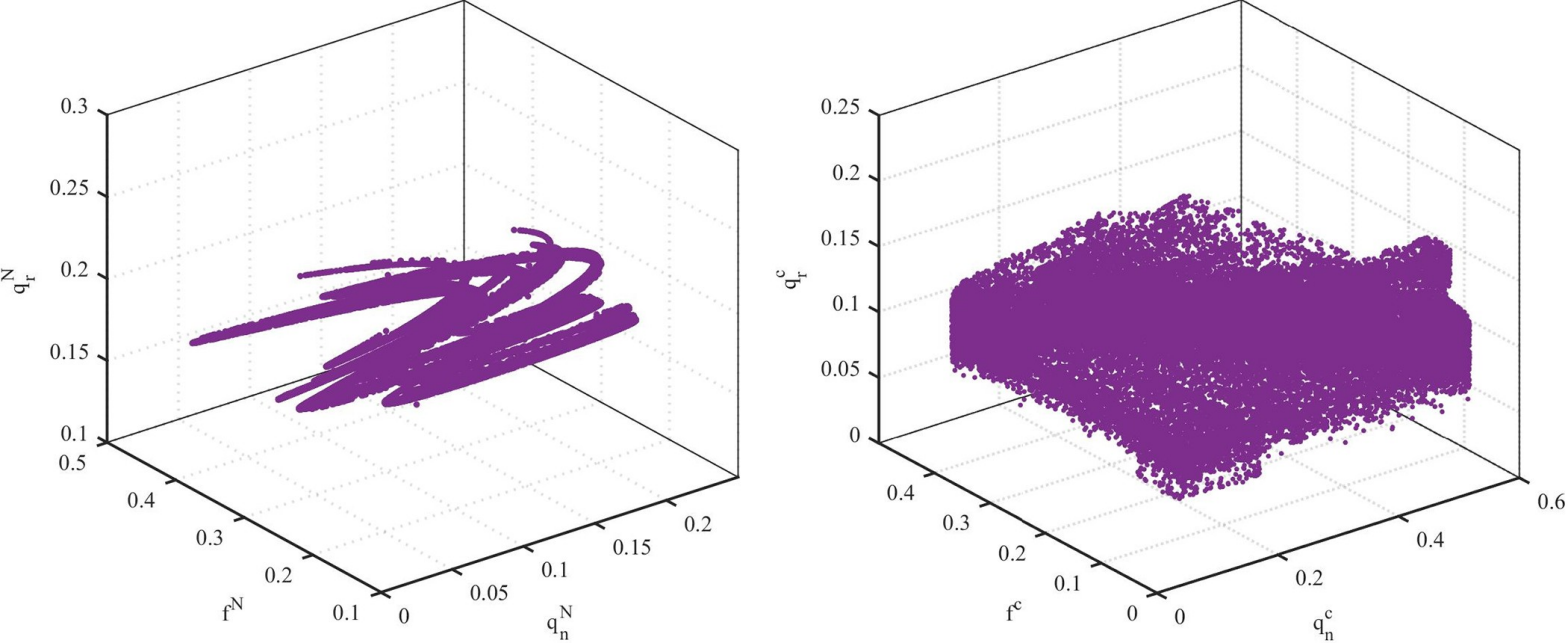
Diagram of attractor: (a) model N with *α*_2_ = 4.5 and *α*_3_ = 7.8. (b) Model C with *α*_2_ = 6.4 and *α*_3_ = 5.5.

Fig 10 shows chaotic attractors in the two models. When the system is in chaos, the number of decision values present in model C (Fig 10(B)) is larger than that in model N (Fig 10(A)). Therefore, the decision in model C is more complex than that in model N. The new product supply chain in chaos in model C requires more information to make decisions and results in increasing the decision-making cost.

**Fig 11:**
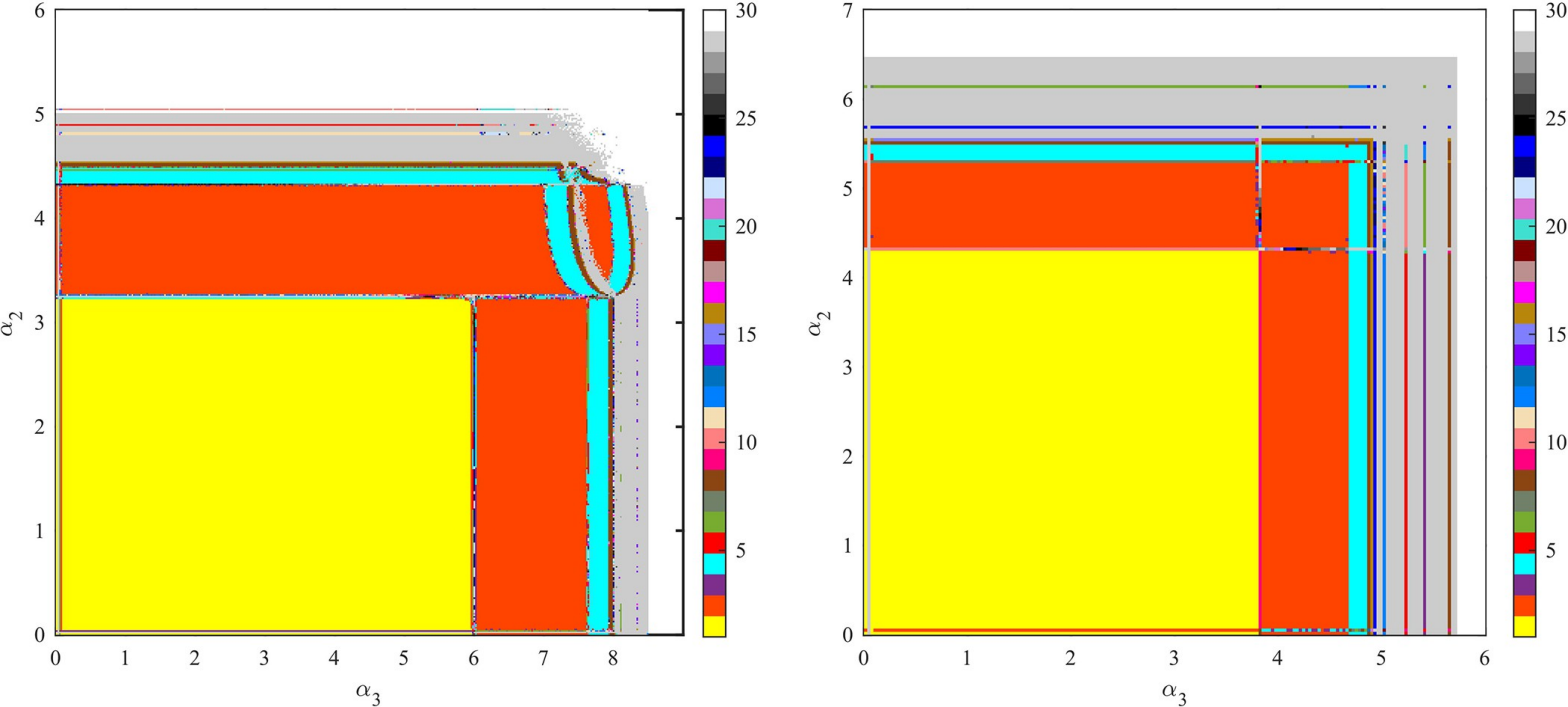
Two-dimensional bifurcation diagram of *α*_2_ and *α*_3_ in (a) Model N and (b) Model C.

Fig 11 shows the two-dimensional bifurcation diagram of the system. Different colors represent the number of available decision values. Yellow means there is only one equilibrium decision value in the system, and orange means two equilibrium decision values. Gray indicates that the system has an infinite number of decision values, but the decision values are distributed as shown in Fig 10. From Fig 11, we observe that the path of the system into chaos in model C is simpler than that in model N.

From Figs 6 to 11, we can see that the stability range of the adjustment parameters of the new product supply chain inventory decreases after the cooperative behavior. When a market with cooperative behavior is chaotic, the volatility of new product inventory is more obvious. However, when cooperative behavior exists, the stability region of the parameter adjustment of the remanufacturing supply chain is significantly improved.

In Section 4.3, we analyze the possible performance of system instability in multi-cycle decision-making.

### 4.3. Average supply chain’s profit in dynamic decision system

**Fig 12:**
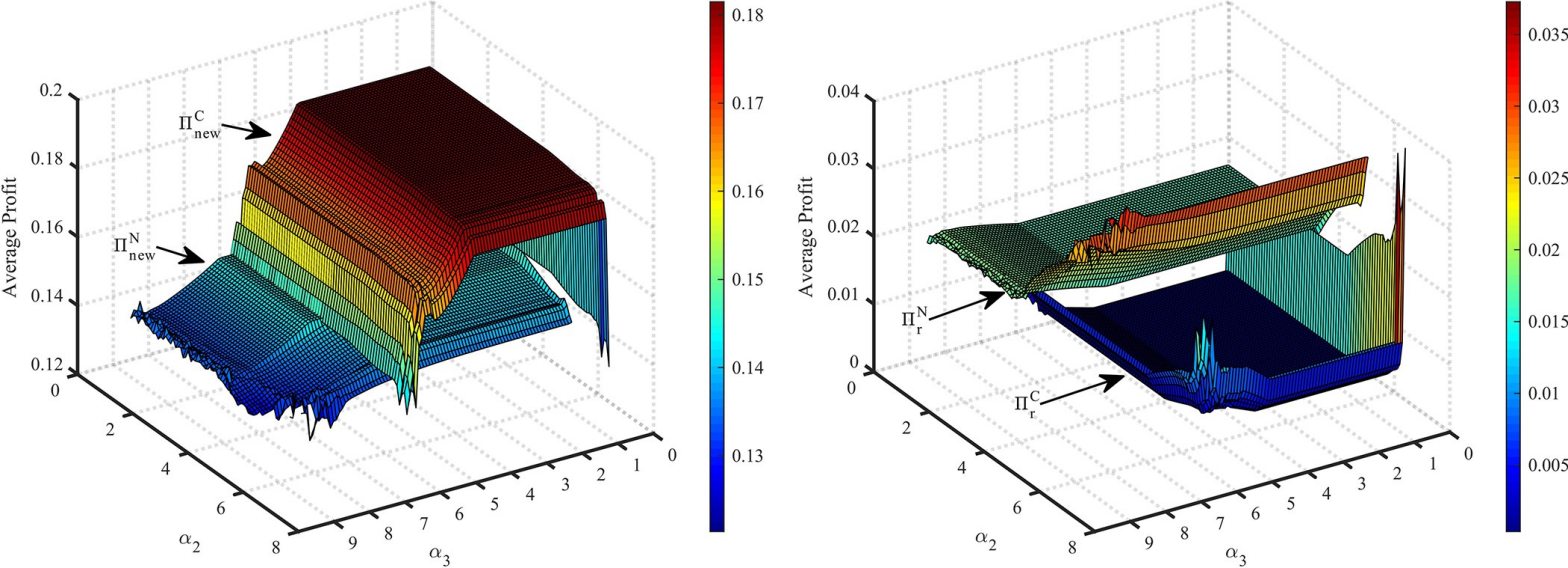
The impact *α*_2_ and *α*_3_ on average profits of supply chain.

Fig 12(A) shows that the equilibrium profit of the new product supply chain in model C is greater than that in model N. In the chaotic state, the supply chain profit of the new products in both models is smaller than the equilibrium profit. When the system is unstable owing to inventory adjustment, the new product supply chain’s profit in model C is more vulnerable to damage.

From Fig 12(B), we can observe that the profit of the remanufactured product supply chain decreases after the new product supply chain cooperation. However, because *α*_2_ and *α*_3_ lead the system into chaos, the remanufacturer may obtain more profit than in the equilibrium decision, and better develop in the market. In particular, in model N, the remanufacturer may benefit more because *α*_2_ leads the system to chaos.

**Fig 13:**
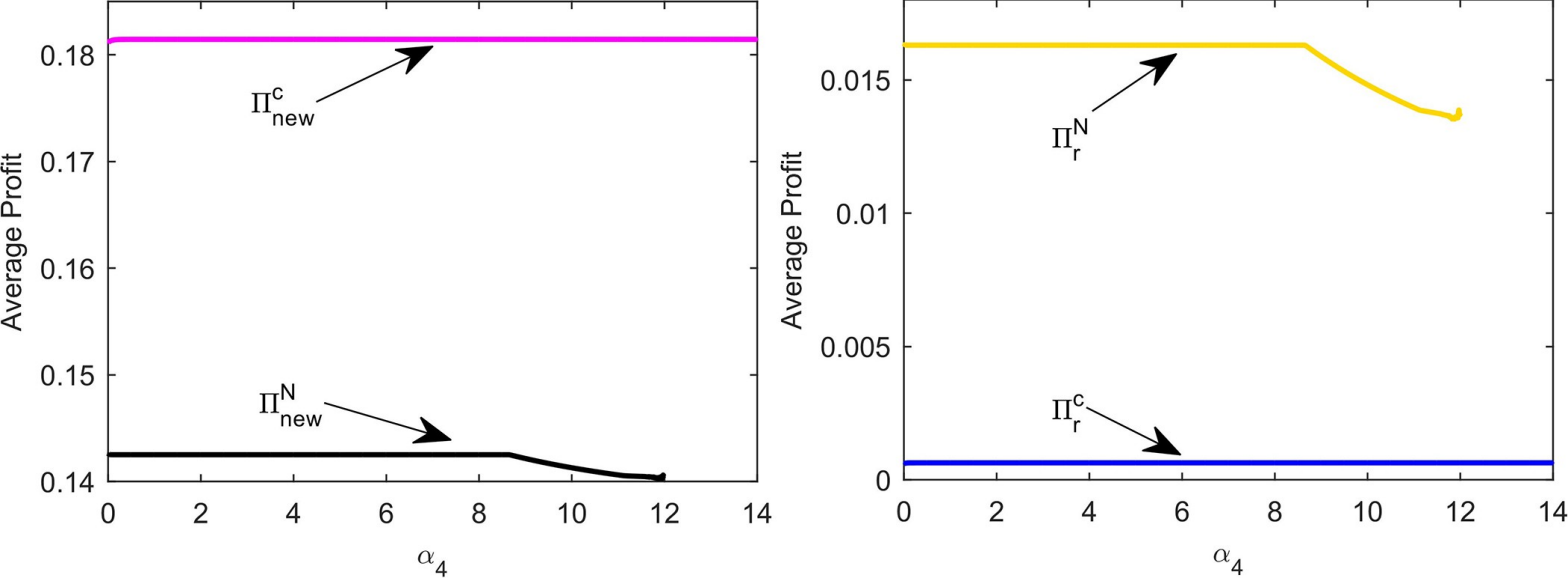
The impact of *α*_4_ on average profits of supply chain.

Fig 13 shows the impact of *α*_4_ on average profit. In model N, because *α*_4_ leads the system into chaos, the profit of both supply chains is lower than the equilibrium profit (black line in Fig 13(A) and yellow line in Fig 13(B)). In model C, the adjustment of *α*_4_ barely affects the profit of the supply chain (magenta line in Fig 13(A) and blue line in Fig 13(B)).

From the above analysis, we can see that in multi-cycle decision-making, both supply chains will lose profits because of improper adjustment of their own decisions. Nevertheless, market followers can reap excess profits from leaders’ volatile decisions. When there is cooperative behavior in the supply chain of new products and the system is in chaos because of the inventory adjustment of new products, the profit of the supply chain of new products may result in a great loss. As long as the remanufactured product supply chain keeps its own decision stability, it may reap stable profits in multi-cycle decision-making.

## 5. Discussion

Based on the theory of nonlinear dynamics, we analyze the influence of the new product supply chain’s cooperative behavior and the complex phenomena that the system may produce in chaos. The main research results are described as follows.

First, the optimal subsidy value of government aiming at social welfare maximization will be significantly affected by cooperative behavior. A possible reason is that the cooperative behavior of the supply chain increases the consumer surplus, and thus, reduces the optimal subsidy. However, the cooperative behavior of supply chain will reduce the profits of the competitive remanufacturing supply chain. The authorized remanufacturing supply chain with cooperative behavior needs more subsidies to develop.

From a dynamic perspective, the effect of increasing government subsidies on market stability under cooperative behavior is smaller than that under non-cooperative behavior. Simultaneously, we find that with the increase of government subsidies to remanufacturers, the decision-making stability range of remanufacturers significantly decreases under cooperative behavior. The profit of the remanufacturers will be much lower than the equilibrium profit after their decisions are unstable.

Based on the above two points, we suggest that when there is cooperative behavior in the supply chain of new products, the government’s subsidy to the authorized remanufacturer should be greater than the optimal subsidy aimed at maximizing social welfare. Nevertheless, subsidized remanufacturers need to reduce the rate of adjustment as subsidies increase.

Further, when cooperative behavior exists in the supply chain of new products, the stable range of inventory decision will decrease. When the market decision system is significantly disturbed, the inventory decision of cooperative supply chain is more likely to lose stability. Cooperative supply chain decision-making in an unstable supply chain is more complicated, and a complex decision system may make the supply chain less resilient [32].

In addition, when the market is unstable due to the inventory decision, the profit of the supply chain of new products will drop significantly. Government subsidies will be conducive to the stability of inventory decisions in the supply chain of new products.

## 6. Directions for future research

By analyzing research issues in social sciences through nonlinear theory, this paper can provide theoretical references for authorized remanufacturing supply chain operation management and government subsidy policy formulation under carbon emission policy from the perspective of system cooperative decision-making. However, we have not considered certain details regarding the supply chain. For example, we did not consider factors, such as the cost of recycling recycled products, altruistic behavior, or interaction between recycled and new product design. In this study, all decisions of the decision-maker were based on the gradient regulation mechanism of the marginal profit of the enterprise, and other bounded rationality regulation mechanisms may produce different results.

Since the development of the authorized remanufacturing closed-loop supply chain is not mature, this paper mainly obtains theoretical results based on highly abstract mathematical models, empirical data and computer experiments. With the development of remanufacturing closed-loop supply chain, theories with more application value can be obtained in the future by means of empirical research based on the combination of market reality data and theoretical results of closed-loop supply chain.

## Supporting information

S1 File (DOCX)
