# Supplementary material for: Complex effects of cooperative behavior on authorized remanufacturing supply chain decisions under subsidies
Source: PLoS One. 2023 Sep 21;18(9):e0291940. doi: 10.1371/journal.pone.0291940 (PMC10513259; doi:10.1371/journal.pone.0291940)
Supplement: S1 File — (DOCX) [file pone.0291940.s001.docx]

S1

Appendix A

In equation (1), the utility of the new product is$\upsilon-p_{n}$. The perceived value of the remanufactured product is$\delta\upsilon$, $0<\delta<1$. The utility of buying the remanufactured product is$\delta\upsilon-p_{r}$. When $\upsilon-p_{n}>\delta\upsilon-p_{r}$ and$\upsilon-p_{n}>0$, the consumers buy the new product and we can deduce $\frac{p_{n}-p_{r}}{1-\delta}<\upsilon<1$; when $\upsilon-p_{n}<\delta\upsilon-p_{r}$ and $\delta\upsilon-p_{r}>0$, the consumers buy the remanufactured product and we can deduce $\frac{p_{r}}{\delta}<\upsilon<\frac{p_{n}-p_{r}}{1-\delta}$. Therefore, we get demand model for $q_{n}$and$q_{r}$: $q_{n}=\int_{\frac{p_{n}-p_{r}}{1-\delta}}^{1} d\upsilon=1-\frac{p_{n}-p_{r}}{1-\delta}$and $q_{r}=\frac{\delta p_{n}-p_{r}}{\delta(1-\delta)}>0$. We can derive equation (1) from $q_{n}$and$q_{r}$.

To ensure the existence of both the new and remanufactured products, $q_{n}=1-\frac{p_{n}-p_{r}}{1-\delta}>0$and $q_{r}=\frac{\delta p_{n}-p_{r}}{\delta(1-\delta)}>0$, we can get $\frac{p_{r}}{p_{n}}<\delta<1-p_{n}+p_{r}$.

Appendix B

$$\begin{matrix} y_{1}=\frac{1}{\delta(\delta-4)}(2c\alpha_{2}-2s\alpha_{2}+16\delta-2\alpha_{1}\delta-2\alpha_{2}\delta-2\alpha_{3}\delta+s\alpha_{3}\delta-2s\alpha_{4}\delta+c\delta(-2\alpha_{1}+\alpha_{3}-\alpha_{4}(\delta-2))-4\delta^{2}+\alpha_{3}\delta^{3}- \\ \alpha_{4}\delta^{2}-ep_{t}\delta(2\alpha_{1}-2\alpha_{3}+\alpha_{4}\delta)+kep_{t}(2\alpha_{2}-\alpha_{3}\delta+2\alpha_{4}\delta)-2\alpha_{2}\varepsilon+\alpha_{3}\delta\varepsilon-2\alpha_{4}\delta\varepsilon) \end{matrix}$$

$$\begin{matrix} y_{2}=\frac{1}{4\delta(\delta-4)^{2}}(-96s\alpha_{2}+16s\alpha_{1}\alpha_{2}+16s\alpha_{2}\alpha_{3}-8s^{2}\alpha_{2}\alpha_{3}+16s^{2}\alpha_{2}\alpha_{4}+384\delta-96\alpha_{1}\delta-96\alpha_{2}\delta+24s\alpha_{2}\delta+ \\ 16\alpha_{1}\alpha_{2}\delta-4s\alpha_{1}\alpha_{2}\delta-96\alpha_{3}\delta+48s\alpha_{3}\delta+16\alpha_{1}\alpha_{3}\delta-8s\alpha_{1}\alpha_{3}\delta+16\alpha_{2}\alpha_{3}\delta-16s\alpha_{2}\alpha_{3}\delta-96s\alpha_{4}\delta+16s\alpha_{1}\alpha_{4}\delta+ \\ 24s\alpha_{2}\alpha_{4}\delta+16s\alpha_{3}\alpha_{4}\delta-8s^{2}\alpha_{3}\alpha_{4}\delta-192\delta^{2}+24\alpha_{1}\delta^{2}+24\alpha_{2}\delta^{2}-4\alpha_{1}\alpha_{2}\delta^{2}+72\alpha_{3}\delta^{2}-12s\alpha_{3}\delta^{2}-8\alpha_{1}\alpha_{3}\delta^{2}-8\alpha_{2}\alpha_{3}\delta^{2}- \\ 48\alpha_{4}\delta^{2}+24s\alpha_{4}\delta^{2}+8\alpha_{1}\alpha_{4}\delta^{2}+8\alpha_{2}\alpha_{4}\delta^{2}+8\alpha_{3}\alpha_{4}\delta^{2}-16s\alpha_{3}\alpha_{4}\delta^{2}+2s^{2}\alpha_{3}\alpha_{4}\delta^{2}+24\delta^{3}-12\alpha_{3}\delta^{3}+12\alpha_{4}\delta^{3}- \\ 6\alpha_{3}\alpha_{4}\delta^{3}+3s\alpha_{3}\alpha_{4}\delta^{3}+\alpha_{3}\alpha_{4}\delta^{4}+c^{2}\left(8\alpha_{2}\left(\alpha_{3}+2\alpha_{4}-\alpha_{4}\delta\right)+4\alpha_{1}\left(\alpha_{2}\left(-4+\delta\right)-2\alpha_{3}\delta+2\alpha_{4}\left(-2+\delta\right)\delta\right)+\alpha_{3}\alpha_{4}\delta\left(8-6\delta+\delta^{2}\right)\right)+ \\ e^{2}pt^{2}(k^{2}\left(-8\alpha_{2}\left(\alpha_{3}-2\alpha_{4}\right)+2\alpha_{3}\alpha_{4}\left(-4+\delta\right)\delta\right)+k\left(16\alpha_{2}\alpha_{3}+4\alpha_{1}\alpha_{2}\left(-4+\delta\right)+8\alpha_{1}\left(\alpha_{3}-2\alpha_{4}\right)\delta-8\alpha_{2}\alpha_{4}\delta+16\alpha_{3}\alpha_{4}\delta-\alpha_{3}\alpha_{4}\delta^{3}\right)+ \\ 2\delta\left(\alpha_{3}\alpha_{4}\left(-4+\delta\right)\delta+\alpha_{1}\left(-8\alpha_{3}+4\alpha_{4}\delta\right)\right))-96\alpha_{2}\varepsilon+16\alpha_{1}\alpha_{2}\varepsilon+16\alpha_{3}\alpha_{2}\varepsilon-16s\alpha_{3}\alpha_{2}\varepsilon+32s\alpha_{2}\alpha_{4}\varepsilon+24\alpha_{2}\delta\varepsilon-4\alpha_{1}\alpha_{2}\delta\varepsilon+48\alpha_{3}\delta\varepsilon- \\ 8\alpha_{1}\alpha_{3}\delta\varepsilon-16\alpha_{3}\alpha_{2}\delta\varepsilon-96\alpha_{4}\delta\varepsilon+16\alpha_{1}\alpha_{4}\delta\varepsilon+24\alpha_{2}\alpha_{4}\delta\varepsilon+16\alpha_{3}\alpha_{4}\delta\varepsilon-16s\alpha_{3}\alpha_{4}\delta\varepsilon-12\alpha_{3}\delta^{2}\varepsilon+24\alpha_{4}\delta^{2}\varepsilon- \\ 16\alpha_{3}\alpha_{4}\delta^{2}\varepsilon+4s\alpha_{3}\alpha_{4}\delta^{2}\varepsilon+3\alpha_{3}\alpha_{4}\delta^{3}\varepsilon-8\alpha_{2}\alpha_{3}\varepsilon^{2}+16\alpha_{2}\alpha_{4}\varepsilon^{2}-8\alpha_{3}\alpha_{4}\delta\varepsilon^{2}+2\alpha_{3}\alpha_{4}\delta^{2}\varepsilon^{2}+ep_{t}(-96\alpha_{1}\delta+16\alpha_{1}\alpha_{2}\delta+96\alpha_{3}\delta- \\ 16\alpha_{2}\alpha_{3}\delta+24\alpha_{1}\delta^{2}-4\alpha_{1}\alpha_{2}\delta^{2}-24\alpha_{3}\delta^{2}-8\alpha_{1}\alpha_{3}\delta^{2}-48\alpha_{4}\delta^{2}+16\alpha_{1}\alpha_{4}\delta^{2}+8\alpha_{2}\alpha_{4}\delta^{2}+12\alpha_{4}\delta^{3}-4\alpha_{3}\alpha_{4}\delta^{3}+\alpha_{3}\alpha_{4}\delta^{4}- \\ 4s\alpha_{1}\left(\alpha_{2}\left(-4+\delta\right)+2\left(\alpha_{3}-2\alpha_{4}\right)\delta\right)+s\alpha_{2}\left(-16\alpha_{3}+8\alpha_{4}\delta\right)+s\alpha_{3}\alpha_{4}\delta\left(-16+\delta^{2}\right)+16\alpha_{1}\alpha_{2}\varepsilon-16\alpha_{2}\alpha_{3}\varepsilon-4\alpha_{1}\alpha_{2}\delta\varepsilon- \\ 8\alpha_{1}\alpha_{3}\delta\varepsilon+16\alpha_{1}\alpha_{4}\delta\varepsilon+8\alpha_{2}\alpha_{4}\delta\varepsilon-16\alpha_{3}\alpha_{4}\delta\varepsilon+\alpha_{3}\alpha_{4}\delta^{3}\varepsilon+4k\alpha_{2}\left(\alpha_{1}\left(-4+\delta\right)+4\alpha_{3}\left(-1+s+\delta+\varepsilon\right)-2\left(-12+4s\alpha_{4}+3\delta+3\alpha_{4}\delta+4\alpha4\varepsilon\right)\right)+ \\ k\delta\left(-8\alpha_{4}\left(2\alpha_{1}+3\left(-4+\delta\right)\right)+\alpha_{3}\left(8\alpha_{1}-\left(-4+\delta\right)\left(-12+\alpha_{4}\left(-4+4s+3\delta+4\varepsilon\right)\right)\right)\right))+c(-4\alpha_{1}\alpha_{2}\left(-4+\delta\right)\left(-1-e\left(1+k\right)p_{t}+s+\delta+\varepsilon\right)+ \\ +8\alpha_{2}(12+2\left(-1+ep_{t}\right)\alpha_{3}-4s\alpha_{4}-3\delta-3\alpha_{4}\delta+s\alpha_{4}\delta+\alpha_{4}\delta^{2}-ep_{t}\alpha_{4}\left(k\left(-4+\delta\right)+\delta\right)-4\alpha_{4}\varepsilon+\alpha_{4}\delta\varepsilon)+ \\ \delta(8\alpha_{1}\left(3\left(-4+\delta\right)+\alpha_{3}\left(1+e\left(-3+k\right)p_{t}-s-\delta-\varepsilon\right)+2\alpha_{4}\left(-1+s-ep_{t}\left(1+k-\delta\right)+\delta+\varepsilon\right)\right)+ \\ \left(-4+\delta\right)\left(12\alpha_{4}\left(-2+\delta\right)+\alpha_{3}\left(-12+\alpha_{4}\left(4+\delta^{2}-ep_{t}\left(4+\left(-3+k\right)\delta\right)+\delta\left(-3+s+\varepsilon\right)\right)\right)\right)))) \end{matrix}$$

$$\begin{matrix} y_{3}=-\frac{1}{2\left(-4+\delta\right)^{3}}(2\left(-4+2s\alpha_{4}+c\alpha_{4}\left(-2+\delta\right)+\delta+\alpha_{4}\delta+ep_{t}\alpha_{4}\left(-2k+\delta\right)+2\alpha_{4}\varepsilon\right) \\ \left(-\left(1+c+ep_{t}\right)\alpha_{1}\alpha_{2}\left(-c-ekp_{t}+s+\delta+\varepsilon\right)+\frac{\left(-4+2\left(1+c+ep_{t}\right)\alpha_{1}+\delta\right)\left(-2c\alpha_{2}-2ekp_{t}\alpha_{2}+2s\alpha_{2}-4\delta+2\alpha_{2}\delta+\delta^{2}+2\alpha_{2}\varepsilon\right)}{\delta}\right)+ \\ 2\left(-4+\delta-\alpha_{3}\left(-2+c-e\left(-2+k\right)p_{t}+s+\delta+\varepsilon\right)\right)\left(\begin{matrix} -\left(1+c+ep_{t}\right)\alpha_{1}\alpha_{2}\left(-c-ekp_{t}+s+\delta+\varepsilon\right) \\ +\frac{\left(-4+2\left(1+c+ep_{t}\right)\alpha_{1}+\delta\right)\left(-2c\alpha_{2}-2ekp_{t}\alpha_{2}+2s\alpha_{2}-4\delta+2\alpha_{2}\delta+\delta^{2}+2\alpha_{2}\varepsilon\right)}{\delta} \end{matrix}\right)+ \\ \left(-4+2\left(1+c+ep_{t}\right)\alpha_{1}+\delta\right)\left(\begin{matrix} \alpha_{3}\alpha_{4}\delta\left(-2+c-e\left(-2+k\right)p_{t}+s+\delta+\varepsilon\right)\left(-c-ekp_{t}+s+\frac{1}{2}\left(1+c+ep_{t}\right)\delta+\varepsilon\right) \\ +2\left(-4+2s\alpha_{4}+c\alpha4\left(-2+\delta\right)+\delta+\alpha_{4}\delta+ep_{t}\alpha_{4}\left(-2k+\delta\right)+2\alpha_{4}\varepsilon\right)\left(-4+\delta-\alpha_{3}\left(-2+c-e\left(-2+k\right)p_{t}+s+\delta+\varepsilon\right)\right) \end{matrix}\right)+ \\ \frac{1}{\delta}\left(-2c\alpha_{2}-2ekp_{t}\alpha_{2}+2s\alpha_{2}-4\delta+2\alpha_{2}\delta+\delta^{2}+2\alpha_{2}\varepsilon\right) \\ \left(\begin{matrix} \alpha_{3}\alpha_{4}\delta\left(-2+c-e\left(-2+k\right)p_{t}+s+\delta+\varepsilon\right)\left(-c-ekp_{t}+s+\frac{1}{2}\left(1+c+ep_{t}\right)\delta+\varepsilon\right)+ \\ 2\left(-4+2s\alpha_{4}+c\alpha_{4}\left(-2+\delta\right)+\delta+\alpha4\delta+ep_{t}\alpha_{4}\left(-2k+\delta\right)+2\alpha_{4}\varepsilon\right)\left(-4+\delta-\alpha_{3}\left(-2+c-e\left(-2+k\right)p_{t}+s+\delta+\varepsilon\right)\right) \end{matrix}\right)) \end{matrix}$$

$$\begin{matrix} y_{4}=\frac{1}{4\left(-4+\delta\right)^{2}\delta}(2s\alpha_{2}+ekp_{t}\left(-2+\alpha_{1}\right)\alpha_{2}+c^{2}\alpha_{1}\alpha_{2}+e^{2}kp_{t}^{2}\alpha_{1}\alpha_{2}-s\alpha_{1}\alpha_{2}-4\delta+2\alpha_{1}\delta+2c\alpha_{1}\delta+2\alpha_{2}\delta\\ -\alpha_{1}\alpha_{2}\delta+\delta^{2}+c\alpha_{2}\left(-2+\alpha_{1}\left(1+e\left(1+k\right)p_{t}-s-\delta-\varepsilon\right)\right)+2\alpha_{2}\varepsilon-\alpha_{1}\alpha_{2}\varepsilon-ep_{t}\alpha_{1}\left(s\alpha_{2}+\left(-2+\alpha_{2}\right)\delta+\alpha_{2}\varepsilon\right)) \\ (\alpha_{3}\left(-2+c-e\left(-2+k\right)p_{t}+s+\delta+\varepsilon\right)\left(-4+2s\alpha_{4}+c\alpha_{4}\left(-2+\delta\right)+\alpha_{4}\delta+ep_{t}\alpha_{4}\left(-2k+\delta\right)+2\alpha_{4}\varepsilon\right) \\ +4\left(-4+2s\alpha_{4}+c\alpha_{4}\left(-2+\delta\right)+\delta+\alpha_{4}\delta+ep_{t}\alpha_{4}\left(-2k+\delta\right)+2\alpha_{4}\varepsilon\right)) \end{matrix}$$

Appendix C

$$u_{1}=\frac{1}{2}\left(-\alpha_{3}\left(-2+c-e\left(-2+k\right)p_{t}+s+\delta+\varepsilon\right)+\frac{\alpha_{2}\left(-c-ekp_{t}+s+\delta+\varepsilon\right)}{\delta}-\frac{2\left(-6+s\alpha_{4}+c\alpha_{4}\left(-1+\delta\right)+3\delta+ep_{t}\alpha_{4}\left(-k+\delta\right)+\alpha_{4}\varepsilon\right)}{-2+\delta}\right)$$

$$\begin{matrix} u_{2}=\frac{\left(c\alpha_{2}+ekp_{t}\alpha_{2}-s\alpha_{2}+2\delta-\alpha_{2}\delta-\alpha_{2}\varepsilon\right)\left(2+\alpha_{3}\left(-2+c-e\left(-2+k\right)p_{t}+s+\delta+\varepsilon\right)\right)}{4\delta}+ \\ \frac{\left(-2+s\alpha_{4}+c\alpha_{4}\left(-1+\delta\right)+\delta+ep_{t}\alpha_{4}\left(-k+\delta\right)+\alpha_{4}\varepsilon\right)\left(2+\alpha_{3}\left(-2+c-e\left(-2+k\right)p_{t}+s+\delta+\varepsilon\right)\right)}{2\left(-2+\delta\right)}+ \\ \frac{\left(c\alpha_{2}+ekp_{t}\alpha_{2}-s\alpha_{2}+2\delta-\alpha_{2}\delta-\alpha_{2}\varepsilon\right)\left(-2+s\alpha_{4}+c\alpha_{4}\left(-1+\delta\right)+\delta+ep_{t}\alpha_{4}\left(-k+\delta\right)+\alpha_{4}\varepsilon\right)}{2\left(-2+\delta\right)\delta} \end{matrix}$$

$$u_{3}=\frac{\left(-c\alpha_{2}-ekp_{t}\alpha_{2}+s\alpha_{2}-2\delta+\alpha_{2}\delta+\alpha_{2}\varepsilon\right)\left(-2+s\alpha_{4}+c\alpha_{4}\left(-1+\delta\right)+\delta+ep_{t}\alpha_{4}\left(-k+\delta\right)+\alpha_{4}\varepsilon\right)\left(2+\alpha_{3}\left(-2+c-e\left(-2+k\right)p_{t}+s+\delta+\varepsilon\right)\right)}{4\left(-2+\delta\right)\delta}$$
